# Biventricular vs. right ventricular pacing devices in patients anticipated to require frequent ventricular pacing (BioPace)

**DOI:** 10.1093/europace/euaf029

**Published:** 2025-03-19

**Authors:** Reinhard C Funck, Hans-Helge Müller, Maurizio Lunati, Luc De Roy, Norbert Klein, Eckhard Meisel, Goran Milasinovic, Mark D Carlson, Michael Wittenberg, Gerhard Hindricks, Jean-Jacques Blanc

**Affiliations:** Klinik für Innere Medizin - Kardiologie, Philipps-Universität Marburg, Baldingerstr, Marburg 35033, Germany; Institut für Medizinische Bioinformatik und Biostatistik, Philipps-Universität Marburg, Hans-Meerwein-Str. 6, Marburg 35043, Germany; Cardiologia 3 – Elettrofisiologia, Ospedale Niguarda, Piazza Ospedale Maggiore 3, Milano 20162, Italy; Cardiology Department, CHU Namur, Ave G. Therasse 1-8, Yvoir 5530, Belgium; Department of Cardiology, Klinikum St. Georg gGmbH, Delitzscher Str. 141, Leipzig 04129, Germany; Praxisklinik HerzKreislauf am WASA-Platz, August-Bebel-Str. 33, Dresden 01219, Germany; Pacemaker Clinic, Klinicki Centar Srbije, Beograd 11000, Serbia; Abbott Cardiac Arrhythmias and Heart Failure, 15900 Valley View Court, Sylmar, CA, USA; Coordinating Center for Clinical Trials, Philipps-University Marburg, Karl-von-Frisch-Str. 4, Marburg 35043, Germany; Department of Cardiology, Angiology and Intensive Care Medicine, Campus Charité Mitte, Charitéplatz 1, Berlin 10117, Germany; Department of Cardiology, Brest University Hospital, Boulevard Tanguy Prigent, Brest 29609, France

**Keywords:** Cardiac pacing, Right ventricular pacing, Pacing-induced cardiomyopathy, Biventricular pacing, Heart failure, Mortality

## Abstract

**Aims:**

Right ventricular (RV) pacing may promote left ventricular (LV) dysfunction. Particularly in patients with preserved LV ejection fraction (LVEF), narrow QRS, and anticipated high ventricular pacing burden (HVPB), evidence is missing that biventricular (BiV) pacing can improve clinical outcome. We therefore evaluated whether implantation of a BiV pacing device (BiVPD) compared with a RV pacing device (RVPD) may improve clinical outcome in predominantly this kind of patients.

**Methods and results:**

In the **B**iventricular Pac**i**ng for atri**o**ventricular Block to **P**revent C**a**rdia**c** D**e**synchronization (BioPace) trial [multicentre, single-blinded (patients), randomized, parallel group], patients were equally allocated to either receive a BiVPD or a RVPD. Co-primary endpoints were (i) the composite of time to death or first heart failure hospitalization and (ii) survival time. We analysed 1810 randomized patients (median age: 73.5 years; female sex: 31.7%; mean LVEF 55.4%; mean QRS 118.4 ms), 902 to BiV and 908 to RV pacing. During mean follow-up of 68.8 months, the difference in the primary composite endpoint between both groups [346 vs. 363 events, hazard ratio (HR) 0.878; 95% confidence interval (CI) 0.756–1.020; *P* = 0.0882) or in mortality (305 vs. 307 deaths, HR 0.926; 95% CI 0.789-1.088; *P* = 0.3492) was smaller than 20%.

**Conclusion:**

In patients, predominantly with preserved LVEF, narrow QRS, and HVPB, superiority of implanting BiVPDs compared with RVPDs could not be proven. Right ventricular pacing may be less harmful for this kind of patients than often suggested and primary BiV pacing does not clearly improve their clinical outcome.

**Clinical trial registration:**

Registered in ClinicalTrials.gov, number NCT00187278 (https://clinicaltrials.gov/ct2/show/study/NCT00187278).

## Graphical Abstract

**Graphical Abstract:**
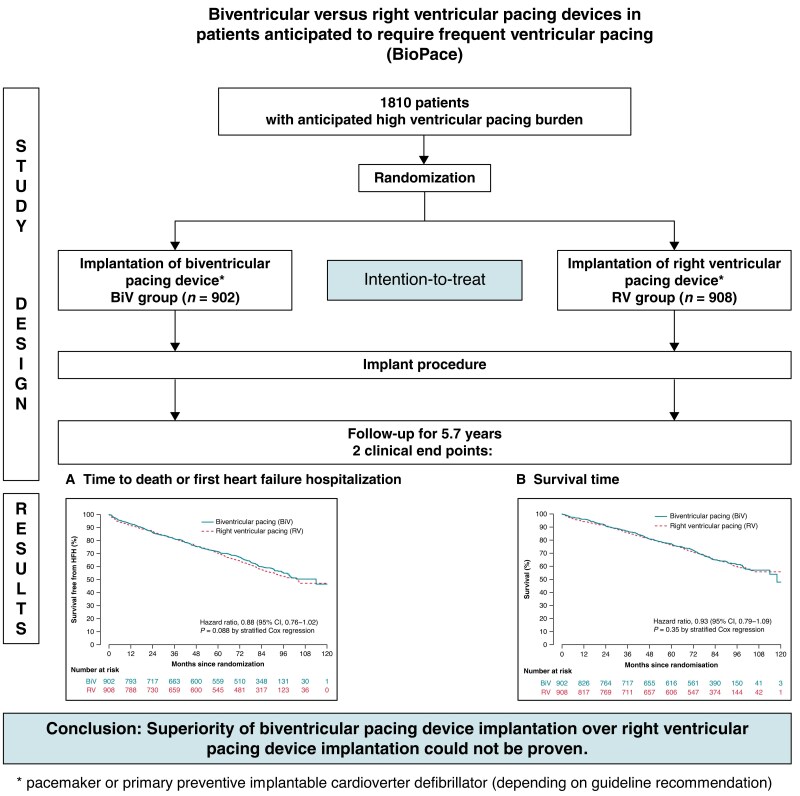

**What’s new?**

- To investigate clinical endpoints, we performed an intention-to-treat analysis on 1810 patients anticipated to have a high ventricular pacing burden, most of them with preserved left ventricular (LV) ejection fraction (LVEF) and narrow QRS, who were randomized to the implantation of a biventricular (BiV) or a right ventricular (RV) pacing device.
- Hardware randomization prior to device implantation and including the implantation procedure of RV and BiV devices in a rather early phase of LV lead implantation in the analysis are stand-alone criteria of our study in the field of pacing-induced cardiomyopathy.
- The study was designed to be as close as possible to real-world clinical practice in that the usual heterogeneity of patients with anticipated high ventricular pacing burden was largely mirrored in the study.
- Our results do not prove superiority of preventive BiV device implantation compared with RV devices in the reduction of the clinical endpoints: (i) death or heart failure hospitalization and (ii) survival time.

## Introduction

Right ventricular (RV) pacing is the current standard for patients with symptomatic atrioventricular block.^1^ Although this pacing modality achieves the primary goal to treat bradycardia, it may promote left ventricular (LV) dysfunction and heart failure in patients with^2,3^ or without^4–6^ pre-existent reduced LV ejection fraction (LVEF). This effect is attributed to the left bundle branch block-like electrical and mechanical ventricular dyssynchrony induced by RV pacing.^3,7,8^

Cardiac resynchronization therapy imitates the physiological interventricular conduction properties and improves LV function and clinical outcomes in patients with heart failure, impaired LV function, and prolonged QRS duration.^9–15^ Therefore, biventricular (BiV) pacing is recommended for patients with impaired LV function and prolonged QRS duration who require frequent ventricular pacing.^1^

In contrast, as highlighted by current guidelines, it is unknown whether BiV stimulation exerts beneficial effects regarding clinically relevant outcomes in patients who require frequent ventricular pacing and who do not necessarily have clinical signs of heart failure, LV dysfunction, or prolonged QRS before device implantation. Smaller studies have reported a reduction of pacing-induced cardiomyopathy in this patient group,^16–18^ but the impact of preventive BiV pacing compared with RV pacing on relevant clinical outcomes in this population has not yet been studied in an adequately powered trial.

The **B**iventricular pac**i**ng for atri**o**ventricular block to **P**revent c**a**rdia**c** d**e**synchronization study (BioPace)^19^ was designed to evaluate whether the implantation of a BiV pacing system is superior to RV pacing regarding the clinical outcomes: (i) time to death or heart failure hospitalization and (ii) survival time in patients anticipated to require frequent ventricular pacing, irrespective of clinical signs of heart failure, LV function, or QRS duration.

## Methods

### Study design

The BioPace study was designed as a single-blind, randomized, controlled, international, multicentre, parallel group study with blinded endpoint assessment. The design of the study has been described previously.^19^ The trial conforms to the Declaration of Helsinki, International Conference on Harmonization of Good Clinical Practice guidelines, and the International Organization for Standardization 14155:2003 and 14155:2011. It was approved by the ethics committee of each participating centre and by the appropriate national and institutional regulatory authorities.

### Patients

We enrolled 1834 patients with Class I indication for a permanent pacing device and an anticipated high ventricular pacing burden irrespective of clinical signs of heart failure, QRS duration, LV size or function, or presence of sinus rhythm. Eligibility criteria have been previously reported^19^ and are listed in *Table 1* and in the Supplementary material online, *Appendix* (Chapter 3). All patients reported for analysis provided written informed consent.

**Table 1:** Inclusion and exclusion criteria

**Inclusion criteria**
1. Presence of an indication for ventricular pacing according to the actual guidelines for the implantation of cardiac pacemakers and a need for frequent (or even permanent) ventricular pacing:
a) permanent third-degree AV block or
b) intermittent third-degree AV block in combination with first-degree AV block with a pQ interval ≥ 220 ms or
c) second-degree AV block Mobitz Type II in combination with first-degree AV block with a pQ interval ≥ 220 ms or
d) second-degree AV block Mobitz Type I (if indicated) in combination with first-degree AV block with a pQ interval ≥ 220 ms or
e) first-degree AV block with a pQ interval ≥ 220 ms and indication for ventricular pacing (includes indication for ventricular pacing based on long HV interval measured during invasive electrophysiological testing) or
f) sick sinus syndrome with symptomatic sinus bradycardia or sinus arrest as primary indication for device implantation in combination with long first-degree AV block with a pQ interval ≥ 220 ms or
g) chronic (permanent) atrial fibrillation (flutter or tachycardia) with a spontaneous heart (ventricular) rate at rest ≤ 60/min or
h) chronic (permanent) atrial fibrillation (flutter or tachycardia) with a spontaneous heart (ventricular) rate at rest ≤ 75/min, if initiation or increase of pharmacological treatment with a relevant heart rate lowering effect (negative chronotropic effect) is planned for the time after pacemaker implantation (i.e.: ß-blockers for heart failure and rate control)
i) patients scheduled for AV node ablation
2. Any QRS duration and morphology
3. LVEF (LVEF without limit) as measured by echocardiography (in at least one plane, either four- or two-chamber or apical long-axis view)
4. Signed written informed consent of the patient or a first-degree relative for study participation after information of the patient/relative about the risks and the aim of the study and willingness and ability to comply with the prescribed follow-up tests and schedule of evaluations
5. Absence of an implanted ventricular pacing device (patients with atrial pacemakers and new need for ventricular pacing may be included)
**Exclusion criteria**
1. ICD or consideration for implantation of an ICD due to arrhythmia indication. However, ICD implant for primary prevention of sudden cardiac death in patients with LVEF ≤ 35% [in accordance with the actual guidelines for the implantation of arrhythmia devices (LVEF ≤ 30%) and in accordance with the results of the SCD-Heft study (LVEF ≤ 35%)] will be allowed
2. Implanted ventricular pacing device
3. Status 1 for cardiac transplantation and likelihood to receive transplantation within 2 years (these patients would not be expected to fulfil the follow-up requirements as outlined in this protocol)
4. Evidence of acute left ventricular dysfunction and high probability for its reversibility (e.g. acute myocarditis and tachy-cardiomyopathy)
5. Implanted prosthetic tricuspid valve
6. Severe musculoskeletal disorder(s)
7. Age below 18 years
8. Current or planned pregnancy in the next 6 months
9. Current or recent (within the past 30 days) participation in any other clinical investigation
10. Life expectancy of less than 6 months
11. Patient´s inability to independently comprehend and complete the quality of life questionnaire

AV, atrioventricular; HV, high ventricular; ICD, implantable cardioverter defibrillator; LVEF, left ventricular ejection fraction.

### Evolution of the study protocol

The initial version of the trial protocol intended to recruit patients with impaired LV function (LVEF ≤ 45%). Due to slow recruitment and emerging data concerning deterioration of initially normal ventricular pump function due to frequent RV stimulation, the protocol was revised by the steering committee in 2004 to include also patients with normal LVEF and further to allow defibrillator implantation in patients with heart failure.^20^ Since patients with normal LV function and hence low expected mortality and hospitalization risk became thus eligible for enrolment, the study sample size was increased from 1200 to 1800 patients and the follow-up duration was prolonged until the observation of 635 deaths. As it was then expected that the study would primarily include patients with preserved LV function, heart failure-specific functional outcomes including functional capacity and quality of life were eliminated as primary endpoints. The composite of death or first heart failure-induced hospitalization was added as a co-primary endpoint to enable the detection of manifest heart failure. During the entire course of the trial, the steering committee was unaware of interim results of any outcome or patient characteristics grouped by treatment arm. Protocol amendments are described in detail in the Supplementary material online, *Appendix* (Chapter 3).

### Randomization and masking

Eligible patients were assigned to BiV or RV pacing in a 1:1 ratio prior to device implantation. Dynamic randomization to balance treatment assignment was centrally provided by the Coordinating Center for Clinical Trials of the Philipps-University Marburg. Participants were blinded regarding the result of randomization and the implanted device.

### Procedures

Procedures for balanced randomization and adjustments in statistical analysis were adapted according to protocol amendments. Depending on atrial rhythm and in accordance with the guidelines,^21^ patients received devices with or without an atrial lead. Implanted devices and leads have been specified earlier.^19^ Left ventricular leads were sought to be implanted in lateral and posterolateral veins. Right ventricular leads were allowed in the apex and in alternative positions. Methods to optimize atrioventricular synchrony as well as medical therapy were left to the discretion of the treating cardiologist and optimal medical therapy for heart failure was encouraged. Pre-defined criteria for protocol-conform switches from RV to BiV pacing were either three hospitalizations due to deterioration of heart failure into New York Heart Association Class IV or the need for intravenous inotropic support.

Follow-up after device implantation consisted of in-clinic visits at Months 1, 3, 6, and 12 and every 12 months thereafter, and telephone follow-up every 6 months between the in-clinic visits to explore patients’ vital status and to collect information about hospitalizations and adverse events.

### Outcomes

There were two co-primary endpoints: (i) composite of time to death or heart failure hospitalization and (ii) survival time. Pre-defined secondary endpoints included time to cardiovascular death or first heart failure hospitalization, time to cardiovascular death, success of LV lead implantation, functional capacity at 12 and 24 months assessed by 6-min walk test, quality of life at 12 and 24 months assessed by the Minnesota Living with Heart Failure Questionnaire, number, incidence, and duration of hospitalizations for heart failure, for cardiovascular events, and for any reason, number and incidence of deaths for heart failure, for cardiovascular events, and for any reason, conversion to permanent atrial fibrillation, adverse events of the implantation procedure, the LV lead, and all leads. *Post hoc* defined endpoints included time to heart failure manifestation leading to death or hospitalization, ventricular pacing percentage, functioning of LV leads, and rate of pacing system infections.

### Statistical analysis

The trial was designed to have 80% power for a two-sided log-rank test at Type I error level of 5% to detect a risk reduction by BiV pacing to 0.8 in terms of the hazard ratio (HR) relative to RV pacing for each of the primary time-to-event endpoints, requiring the observation of 635 events. The Type I error for multiple testing was strongly controlled at the significance level of 5% by testing hierarchically. It was planned to test first the composite endpoint after 635 composite events. In case of significance, it was intended to test survival time after 635 deaths would have been observed. Incorporating prognostic factors, the confirmatory analysis used the pre-defined 16 strata (see Supplementary material online, *Appendix*, Chapter 3) and age for additional adjustment within stratified Cox regression. The number of 635 events of the composite primary endpoint was expected to be observed by the end of May 2014. When design adaptations were made, Type I error was controlled respecting the conditional rejection probability principle.^22,23^

The time-to-event endpoints were analysed on an intention-to-treat basis using the Kaplan–Meier method and applying the stratified log-rank test and the stratified Cox proportional hazard models to test differences and to estimate the HRs with 95% confidence intervals (CIs) for comparison of therapy groups. The confirmatory analysis comprises the two co-primary endpoints. For the description of quantitative traits, the mean ± standard deviation (SD) was provided, and group differences were evaluated using the *t*-test except for traits with skewed distribution where the Wilcoxon–Mann–Whitney test was applied. For categorical traits, Fisher’s exact test was used to evaluate group differences.

Implant failures and the functional status of the implanted devices at 3 months after initial implantation were described according to BiV or RV implantation attempts. Causes of death and hospitalizations were adjudicated by an independent endpoint adjudication committee. An independent Data Safety Monitoring Board monitored the conduct of the trial.

Analyses were performed with SAS, versions 9.3 and 9.4 (SAS Institute, Cary, NC, USA), StatXact of Cytel Studio, version 6.3.0 (Cytel Studio; Cytel Corporation, Cambridge, MA, USA), and the R Project for Statistical Computing.

The study is registered with the ClinicalTrials.gov identifier: NCT00187278.

### Role of the funding source

The steering committee and the study statistician designed the study and wrote the study protocol in cooperation with representatives of the Center for Clinical Trials (KKS) in Marburg and the funder. The latter performed clinical monitoring, data collection, and reporting of safety issues. Randomization, data management, and analysis were centrally performed by the KKS.

The members of the steering committee and of the Data Safety Monitoring Board, the involved collaborators of the KKS, and representatives of the funder had full access to the data, and all authors had final responsibility for the decision to submit for publication.

## Results

**Figure 1:**
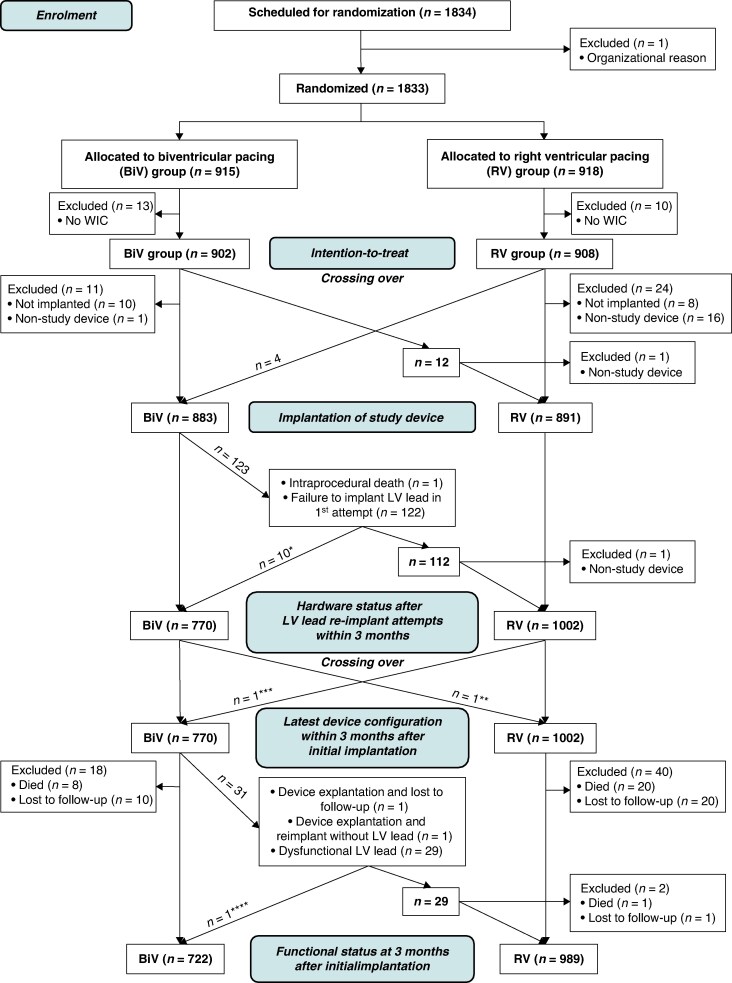
Trial profile representing patient randomization, device implantation, re-implant attempts, hardware status, and functional status at 3 months after initial implant. * Successful LV lead implant during second attempt within 3 months after initial implant attempt. ** LV lead deactivated right after implantation (patient received BiV device despite having been randomized to RV). *** Upgrade to BiV within first 3 months after initial implant (physician´s decision). **** Loss of left ventricular capture and re-implantation of LV lead 2 days after initial implantation. BiV, biventricular; LV, left ventricular; RV, right ventricular; WIC, written informed consent.

From May 2003 to September 2007, 1833 patients were randomized in 94 hospitals in 15 countries: 915 to BiV and 918 to RV pacing. Due to missing written informed consent, a total of 902 patients were finally analysed in the BiV and 908 in the RV pacing arm (*Figure 1*). Among baseline characteristics, we observed advanced age (73.5 ± 9.2 years), 31.7% females, preserved LVEF (55.4 ± 12.2%), and mostly a narrow QRS complex (118.4 ± 30.5 ms). Baseline characteristics and indications for permanent pacing are shown in *Table 2*. Patients were followed until the end of May 2014 (68.8 months mean follow-up).

**Table 2:** Baseline characteristics and indications for device implantation

Characteristic	All patients (*n* = 1810)	RV pacing (*n* = 908)	BiV pacing (*n* = 902)
Age—year	73.5 ± 9.2	73.3 ± 9.3	73.8 ± 9.0
Men—no. (%)	1236 (68.3)	612 (67.4)	624 (69.2)
Body mass index—kg/m^2^	27.4 ± 4.5	27.5 ± 4.7	27.4 ± 4.3
Ventricular pacing at 1 month—%^a^	88.3 (56, 91, 99)	86.4 (48, 88, 99)	90.2 (67, 93, 99)
Ventricular pacing at 1 month ≥ 90%—no. (%)	1249 (77.1)	592 (73.7)	657 (80.3)
Underlying cardiac diseases—no. (%)	1143 (63.1)	572 (63.0)	571 (63.3)
Ischaemic heart disease	548 (30.3)	274 (30.2)	274 (30.4)
Hypertensive heart disease	543 (30.0)	281 (30.9)	262 (29.0)
Dilated cardiomyopathy	94 (5.2)	43 (4.7)	51 (5.7)
Valvulopathy	335 (18.5)	170 (18.7)	165 (18.3)
Prior MI	308 (17.0)	162 (17.8)	141 (15.6)
Cardiovascular risk factors—no. (%)			
Hypertension	1362 (75.3)	696 (76.7)	666 (73.8)
Diabetes mellitus	506 (28.0)	255 (28.1)	251 (27.8)
Left ventricular ejection fraction (LVEF)—%	55.4 ± 12.2	55.5 ± 12.4	55.3 ± 12.1
Patients with—no. (%)			
LVEF ≤ 35%	152 (8.4)	74 (8.1)	78 (8.6)
35% < LVEF ≤ 50%	419 (23.1)	221 (24.3)	198 (22.0)
LVEF > 50%	1239 (68.5)	613 (67.5)	626 (69.4)
QRS duration—ms	118.4 ± 30.5	118.8 ± 30.3	118.1 ± 30.8
Patients with—no. (%)			
QRS ≤ 120 ms	1142 (63.1)	571 (62.9)	571 (63.3)
120 ms < QRS ≤ 150 ms	366 (20.2)	190 (20.9)	176 (19.5)
QRS > 150 ms	302 (16.7)	147 (16.2)	155 (17.2)
Left bundle branch block—no. (%)	316 (17.5)	166 (18.3)	150 (16.6)
Atrial arrhythmias—no. (%)	450 (24.9)	225 (24.8)	225 (24.9)
Scheduled for ICD—no. (%)	48 (2.7)	22 (2.4)	26 (2.9)
Indications for device implantation—no. (%)			
Persistent bradycardia	1121 (61.9)	568 (62.6)	553 (61.3)
Advanced AVB^b^	822 (45.4)	421 (46.4)	401 (44.5)
Atrial tachyarrhythmias with symptomatic slow ventricular rate^c^	299 (16.5)	147 (16.2)	152 (16.9)
Intermittent bradycardia	687 (38.0)	338 (37.2)	349 (38.7)
Paroxysmal second- or third-degree AVB with long PR interval^d^	465 (25.7)	228 (25.1)	237 (26.3)
Symptomatic SSS with long PR interval	222 (12.3)	110 (12.1)	112 (12.4)
Unknown	2 (0.1)	2 (0.2)	0 (0.0)

Presentation of characteristics: quantitative as mean ± SD, categorical as no. (%).

AVB, atrioventricular block; BiV, biventricular; ICD, implantable cardioverter defibrillator; MI, myocardial infarction; RV, right ventricular; SSS, sick sinus syndrome.

^a^Mean (10th percentile, 1st quartile, median) since distribution is skewed towards lower values.

^b^Includes patients with permanent third-degree AVB, with second-degree Type II AVB with PR interval ≥ 220 ms, or with AV node ablation.

^c^Includes patients with atrial fibrillation and flutter with resting ventricular rates ≤ 60 bpm or with resting ventricular rates ≤ 75 bpm and need for negative chronotropic therapy.

^d^Includes patients with first-degree AVB with PR interval ≥ 220 ms and indication for ventricular pacing such as long HV interval measured during invasive electrophysiological testing.

Devices were implanted a median of 2 and 1 days after randomization in the BiV and RV pacing arm, respectively. In six patients, initial implantation attempts were made between 99 and 161 days post-randomization. One hundred sixty-six single-chamber [18.6%, 163 pacemakers and 3 implantable cardioverter defibrillators (ICDs)] and 725 dual-chamber (81.4%, 702 pacemakers and 23 ICDs) RV pacing study devices were successfully implanted in 891 patients. Of those, 880 had initially been randomized to RV and 11 to BiV pacing. Biventricular study devices were implanted successfully in the first attempt in 760 of 883 patients (86.1%). Of these 883 patients, 879 had initially been randomized to BiV and 4 to RV pacing. In 10 patients, the LV lead was implanted in a second attempt within three months after the first attempt (87.2% overall BiV implant success rate). Within the first 3 months after successful BiV implantation, the BiV device was explanted due to infection in one patient, and in another patient, the initially implanted BiV device was downgraded to RV pacing due to LV lead dislodgement. During the same period, LV lead dysfunctions were documented in 29 patients (3.8%). At 3 months post-implant, 722 and 989 patients with study devices were functionally on effective BiV and RV pacing, respectively (*Figure 1*; Supplementary material online, *Appendix*, Chapter 8.1).

Later than 3 months after initial implantation, there were 55 crossovers from BiV to RV pacing and 52 crossovers from RV to BiV pacing (see Supplementary material online, *Table S6*, for further details; see also Supplementary material online, *Appendix*). The crossovers from BiV to RV pacing were mainly due to implant failures and dysfunctional LV leads. Of the 52 crossovers from RV to BiV pacing that occurred later than 3 months after initial implantation, 6 patients had initially been randomized to BiV pacing, 3 were protocol-conform switches, 14 were clinically indicated according to the IEAC, and 29 occurred at physician’s discretion. In total, 258 patients of the intention-to-treat cohort (14.3%) crossed over, 208 (23.1%) from BiV to RV pacing and 50 (5.5%) vice versa.

In both study arms and irrespective of LVEF, most RV leads were implanted in the RV apex (>80%). Septal positions were slightly more frequent in the BiV arm than in the RV arm (*Table 3*). In total, 51 and 49 patients were lost to follow-up in the BiV and in the RV group, respectively.

**Table 3:** Right ventricular lead positions in both arms relative to LVEF ≤50% and >50%

Lead pos.	BiV (*n* = 902)		RV (*n* = 908)	
	LVEF ≤ 50% (*n* = 306)	LVEF > 50% (*n* = 596)	LVEF ≤ 50% (*n* = 308)	LVEF > 50% (*n* = 600)
Apical	247 (80.7%)	487 (81.7%)	264 (85.7%)	530 (88.3%)
Septal	37 (12.1%)	72 (12.1%)	28 (9.1%)	42 (7.0%)
Other pos.	14 (4.6%)	29 (4.9%)	12 (3.9%)	20 (3.3%)
Not implanted/non-study device etc.	8 (2.6%)	8 (1.3%)	4 (1.3%)	8 (1.3%)

BiV, biventricular; RV, right ventricular; LVEF, left ventricular ejection fraction.

### Primary endpoints

**Figure 2:**
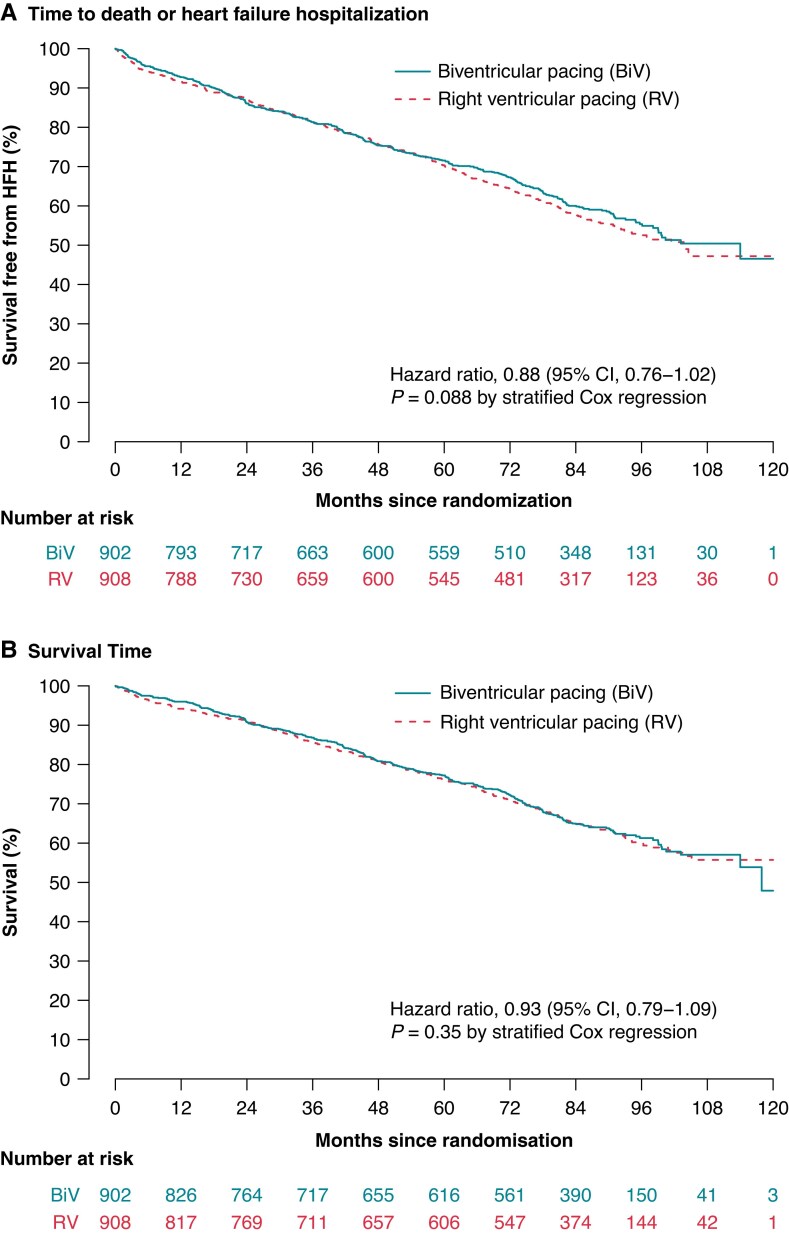
(*A*) Time to death or first hospitalization. (*B*) Survival time. Kaplan–Meier estimates are shown for the comparison of patients with RV and BiV pacing. BiV, biventricular; CI, confidence interval; RV, right ventricular.

Co-primary composite endpoint events occurred in 709 patients (death in 449 patients and first heart failure hospitalization in 260 patients), namely in 346 of 902 patients (38.4%) assigned to the BiV pacing arm (death in 226 patients and first heart failure hospitalization in 120 patients) and in 363 of 908 patients (40.0%) assigned to RV pacing (death in 223 patients and first heart failure hospitalization in 140 patients). The HR was 0.878 (95% CI 0.756–1.020) without statistical significance between both study arms (*P* = 0.0882; *Figure 2A*; *Table 4*; Supplementary material online, *Figure S1*).

**Table 4:** Primary, secondary, and explanatory time to event outcomes

Outcome	BiV pacing (*n* = 902)			RV pacing (*n* = 908)			Hazard ratio (95% CI)				*P*-value
	LVEF subgroup			LVEF subgroup			LVEF subgroup			All	
	≤35% (*n* = 78)	36%─50% (*n* = 198)	>50% (*n* = 626)	≤35% (*n* = 74)	36%─50% (*n* = 221)	>50% (*n* = 613)	≤35% (*n* = 152)	36%─50% (*n* = 419)	>50% (*n* = 1239)	(*n* = 1810)	
	No. of patients with events (%)^a^										
Primary outcomes											
Time to death	46 (59.0)	75 (37.9)	184 (29.4)	39 (52.7)	81 (36.7)	187 (30.5)	1.09 (0.69─1.71)	0.92 (0.67─1.26)	0.90 (0.74─1.11)	0.93 (0.79─1.09)	0.3492
Time to death or hospitalization due to heart failure	51 (65.4)	88 (44.4)	207 (33.1)	48 (64.9)	102 (46.2)	213 (34.7)	0.83 (0.54─1.26)	0.88 (0.66─1.17)	0.90 (0.74─1.08)	0.88 (0.76─1.02)	0.0882
Secondary outcomes											
Time to cardiovascular death	23 (29.5)	26 (13.1)	58 (9.3)	17 (23.0)	31 (14.0)	58 (9.5)	1.31 (0.67─2.55)	0.85 (0.50─1.43)	0.94 (0.65─1.36)	0.97 (0.74─1.27)	0.8215
Time to cardiovascular death or hospitalization due to heart failure	33 (42.3)	51 (25.8)	96 (15.3)	32 (43.2)	62 (28.1)	103 (16.8)	0.79 (0.47─1.33)	0.85 (0.58─1.23)	0.88 (0.67─1.17)	0.86 (0.70─1.05)	0.1394
Explanatory outcome											
Time to heart failure manifestation leading to death or hospitalization	30 (38.5)	40 (20.2)	68 (10.9)	32 (43.2)	52 (23.5)	76 (12.4)	0.75 (0.44─1.27)	0.81 (0.54─1.23)	0.84 (0.61─1.17)	0.81 (0.65─1.03)	0.0817

BiV, biventricular; LVEF, left ventricular ejection fraction; RV, right ventricular.

^a^Percentage from every subgroup.

In the second co-primary endpoint survival time, 612 deaths were observed, 305 of 902 patients (33.8%) assigned to BiV pacing, and 307 of 908 (33.8%) assigned to RV pacing. The HR was 0.926 (95% CI 0.789–1.088), also without statistical significance between both study arms (*P* = 0.3492; *Figure 2B*; *Table 4*; Supplementary material online, *Figure S2*).

Due to the considerable number of crossovers, a sensitivity analysis and an as-treated analysis were performed. They did not reveal relevant differences compared with the intention-to-treat analysis (see Supplementary material online, *Appendix*, Chapter 8.5). Also, the analysis of the subgroup of patients with ventricular pacing percentages of at least 90% at 1 month after device implantation did not deliver substantially different results.

### Secondary endpoints

Regarding time to cardiovascular death, HR was 0.969 (95% CI 0.737–1.274; *Table 4*; Supplementary material online, *Figures S5* and *S6*), and regarding the combined endpoint cardiovascular death or heart failure hospitalization, HR was 0.857 (95% CI 0.697–1.052; *Table 4*; Supplementary material online, *Figures S3* and *S4*). There were almost no differences between both study arms in the distances covered in the 6-min walk test and in the results of the Minnesota Living with Heart Failure Questionnaire (*Table 5*). Time spent in hospital and mortality due to cardiovascular events were also almost the same in both study arms (*Table 5*).

**Table 5:** Secondary endpoints

Characteristic	All patients (*n* = 1810)	RV pacing (*n* = 908)	BiV pacing (*n* = 902)
Dist. covered in 6MWT at 12 months (m)	371.3 ± 128.9/1347	371.3 ± 128.8/672	371.2 ± 129.1/675
LVEF ≤ 35%	345.9 ± 137.6/98	354.9 ± 127.3/51	336.2 ± 148.7/47
35% < LVEF ≤ 50%	357.4 ± 122.8/297	357.6 ± 121.5/155	357.1 ± 124.7/142
LVEF > 50%	378.2 ± 129.3/952	377.7 ± 131.0/466	378.7 ± 127.7/486
Dist. covered in 6MWT at 24 months (m)	372.9 ± 129.6/1206	369.3 ± 127.2/614	376.5 ± 132.0/592
LVEF ≤ 35%	362.4 ± 127.9/73	357.3 ± 136.6/37	367.6 ± 120.0/36
35% < LVEF ≤ 50%	357.0 ± 130.0/262	357.8 ± 131.9/143	356.0 ± 128.3/119
LVEF > 50%	378.5 ± 129.3/871	374.1 ± 124.8/434	382.8 ± 133.6/437
MLWHFQ at 12 months	15.7 ± 15.4/1439	16.0 ± 15.4/724	15.3 ± 15.5/715
LVEF ≤ 35%	21.4 ± 18.0/105	22.0 ± 18.5/54	20.7 ± 17.6/51
35% < LVEF ≤ 50%	17.5 ± 16.9/318	18.0 ± 17.3/168	16.9 ± 16.4/150
LVEF > 50%	14.5 ± 14.5/1016	14.7 ± 14.2/502	14.3 ± 14.8/514
MLWHFQ at 24 months	17.4 ± 17.1/1337	17.5 ± 17.6/662	17.3 ± 16.6/675
LVEF ≤ 35%	21.8 ± 19.0/90	22.4 ± 20.2/40	21.2 ± 18.1/50
35% < LVEF ≤ 50%	20.6 ± 18.1/301	20.6 ± 18.6/158	20.6 ± 17.6/143
LVEF > 50%	15.9 ± 16.3/946	16.0 ± 16.8/464	15.9 ± 15.9/482
Hospitalizations (no. patients at risk)	1787	899	888
Heart failure as primary reason, HHF			
Number of hospitalizations	409	217	192
Incidence (hosp./patient year)	0.040	0.043	0.037
Duration (days/patient year)	0.40	0.40	0.39
Number with unknown reason^a^	39	19	20
Number with unclassifiable reason^a^	28	14	14
Caused by a CVE, HCVE			
Number of hospitalizations	1648	823	825
Incidence (hosp./patient year)	0.162	0.162	0.161
Duration (days/patient year)	1.51	1.52	1.50
Number with unknown reason^a^	39	19	20
Number with unclassifiable reason^a^	28	14	14
Any reason, HAR			
Number of hospitalizations	4199	1987	2212
Incidence (hosp./patient year)	0.412	0.392	0.432
Duration (days/patient year)	3.93	3.72	4.14
Deaths (no. patients at risk)	1810	908	902
Caused by heart failure, DHF			
Number of deaths	110	53	57
Incidence (deaths/patient year)	0.0108	0.0104	0.0111
Number with unclassifiable reason	155	88	67
Caused by a CVE, DCVE			
Number of deaths	213	106	107
Incidence (deaths/patient year)	0.0208	0.0208	0.0208
Number with unknown reason	111	61	50
Number with unclassifiable reason	31	20	11
Any reason, DAR			
Number of deaths	612	307	305
Incidence (deaths/patient year)	0.0598	0.0604	0.0593
Conversion to permanent AF			
Total	167	91	76
6 months	18/1541	4/766	14/775
12 months	23/1497	10/742	13/755
24 months	12/1398	9/695	3/703
36 months	23/1242	11/617	12/625
48 months	24/1073	12/543	12/530
60 months	21/959	11/485	10/474
72 months	23/854	16/416	7/438
84 months	12/546	10/265	2/281
96 months	8/212	7/98	1/114
108 months	3/70	1/38	2/32
120 months	0/4	0/2	0/2

6MWT, 6-min walk test; BiV, biventricular; CVE, cardiovascular event; DAR, death caused by any reason; DCVE, death caused by cardiovascular event; DHF, death caused by heart failure; HAR, hospitalization for any reason; HCVE, hospitalization for cardiovascular event; HHF, hospitalization for heart failure; MLWHFQ, Minnesota Living with Heart Failure Questionnaire; Permanent AF, defined as occurrence of atrial fibrillation after initial, documented by 12-lead ECG implant in two subsequent visits, presentation of tTotal no. of conversions and no. of conversions/no. of patients with rhythm available for each visit where ECG was performed; presentation of 6MWT and MLWHFQ, mean ± SD/no. assessed patients; RV, right ventricular.

^a^The patients with unknown and unclassifiable reasons listed under HHF and HCVE are the same.

### *Post hoc* defined endpoints

Hazard ratio for the explanatory endpoint time to heart failure manifestation resulting in death or hospitalization was 0.814 (95% CI 0.646–1.026; *Table 4*; Supplementary material online, *Figures S7* and *S8*).

Median ventricular pacing percentage was ≥98.0% in both study arms (*Table 2*; Supplementary material online, *Figure S9* and *Table S1*) throughout the study.

Subgroup analyses describing heterogeneity of treatment effects regarding factors like LVEF used for adjustment in time-to-event outcomes can be found in *Table 4* and in Supplementary material online, *Figures S1*, *S2*, *S4*, *S6*, and *S8*.

An analysis of the primary, secondary, and explanatory time-to-event outcomes in the groups of patients with different ranges of LVEF is given in *Table 4*.

Subgroup analyses describing heterogeneity regarding LVEF in ventricular pacing percentage show high ventricular pacing burdens throughout the study period (see Supplementary material online, *Figures S9*–*S12* and *Tables S1*–*S4*).

### Adverse events

A total of 4050 serious adverse events occurred, 2111 in the BiV pacing arm and 1939 in the RV pacing arm, and they appeared more often in patients with an LV lead (see Supplementary material online, *Tables S7* and *S9*). Most of the serious adverse events were hospitalizations. The rate of patients with serious device-related infection was 1.66% (30 of 1810 patients), 1.43% in the RV group (13 patients and 1.88% in the BiV group (17 patients); the absolute risk increased by 0.45% (95% CI −0.72 to 1.63%; *P* = 0.47). Most LV lead-associated adverse events were non-serious (see Supplementary material online, *Tables S8* and *S10*).

## Discussion

The results of our intention to treat trial that included pre-implant hardware randomization, and hence the implant procedure, did not prove superiority of BiV over RV pacing in patients mostly without overt heart failure, with preserved LVEF, normal QRS duration, and high ventricular pacing burden. The effects we observed were smaller than the pre-defined detectable difference of 20% reduction of the hazards to experience (i) the composite of death and first heart failure hospitalization and (ii) death, each with power of 80%. The respective reductions we observed were only 12.2 and 7.4%. Furthermore, analyses of pre-defined subgroups of patients with different ranges of LVEF (*Table 2*) corroborated the findings of the main analysis regarding the primary and secondary outcomes.

Previous studies have demonstrated a superiority of BiV pacing over RV pacing in patients with an indication for permanent ventricular pacing and *impaired* LV function. Based on Bayesian modelling, the BLOCK-HF (Biventricular Pacing for Atrioventricular Block and Systolic Dysfunction) trial^24^ showed a reduction in the composite primary endpoint that consisted of time to death of any cause, an urgent care visit for heart failure that required intravenous therapy, or an at least 15% increase of LV end-systolic volume index, as compared with the value at randomization. This was primarily driven by a reduction of events related to heart failure.

In contrast to patients with *impaired* LV function, indication for cardiac pacing, and anticipated high ventricular pacing burden, there are only very limited data about the potential impact of BiV pacing compared with RV pacing in patients with *preserved* LV function. The PREVENT-HF (preventing ventricular dysfunction in pacemaker)^18^ trial and the PACE (Pacing to Avoid Cardiac Enlargement)^17^ trial compared BiV with RV stimulation in patients with preserved ejection fraction with regard to LV volume and function but not for clinical endpoints. In PREVENT-HF, there was no difference of LV end-diastolic volume between BiV and RV pacing. PACE reported a deterioration of LV function in patients receiving RV stimulation, while ventricular function was mainly preserved in patients with BiV pacing. In this small trial, RV stimulation was also associated with a higher rate of heart failure hospitalizations when compared with BiV pacing.^17^ However, results from large studies based on frequentistic analyses comparing clinical outcomes among patients undergoing BiV or RV pacing in this kind of population are missing, although urgently needed for clinical decision-making in daily practice.

Pacing-induced cardiomyopathy is thought to be a consequence of ventricular dyssynchrony induced by a high burden of RV pacing.^7^ Retrospective studies have investigated the impact of permanent pacing on ventricular function and heart failure symptoms: Permanent RV pacing can potentially deteriorate LV function and increase heart failure hospitalizations.^5–7^ Cardiac resynchronization therapy, on the other hand, may reverse these detrimental effects and could theoretically prevent RV pacing-induced deterioration of LV function and thus potentially improve clinical outcome.^5^ According to the results of our study, it must be concluded that when designing the study, either (i) the beneficial effects of BiV pacing or (ii) the deleterious effects of RV pacing have been overestimated, especially in patients with preserved LVEF. At present, it cannot be concluded that preventive implantation of BiV pacing devices in patients with preserved ejection fraction and anticipated high ventricular pacing burden leads to fewer heart failure hospitalizations or a reduction in mortality when compared with RV stimulation. The negative prognostic implications of RV pacing-induced cardiac desynchronization in patients without LV dysfunction described in other studies might also have been overestimated.^25^ They could at least not be confirmed at the level of important clinical outcome parameters in our large, prospective, randomized trial. Another explanation for the lack of benefit of BiV stimulation may be possible harm induced even by BiV pacing in patients with narrow QRS as has been demonstrated in patients with impaired LV function in the ECHO CRT trial.^26^

Physiologic pacing by either BiV pacing or conduction system pacing such as His bundle pacing^27–31^ or left bundle branch (area) pacing,^32–36^ in patients with reduced LVEF, may result in a better preservation or even in an increase of LV systolic function and haemodynamic parameters.^37^ However, superiority of these ‘physiologic pacing modes’ over RV pacing still remains to be proven, especially in patients with normal LVEF and normal QRS width.

### Study limitations

The study design was adapted several times. This can be considered to be a potential limitation of our study. However, in the context of the scientific results that were published during the course of the trial, we found it reasonable to amend the study in order not to exclude an important subpopulation from our study.

Initially, our goal was to include only patients with LVEF ≤ 45%. However, due to slow recruitment and due to increasing numbers of publications on the disadvantageous effects of high RV pacing burdens even in patients without impaired LVEF, we considered it reasonable to open the study also for patients with preserved LVEF. This induced the increase of the study cohort and of the necessary follow-up duration. We also added the composite death or first hospitalization due to heart failure as second co-primary endpoint. As primary preventive implantation of ICDs in patients with severely impaired LVEF was meanwhile recommended by international guidelines, we also allowed primary preventive implantation of ICDs in those patients, either as BiV or RV devices, depending on randomization.

Whether these amendments are considered to be study limitations is debatable, since certain patient groups and the composite endpoint were added to the study protocol and none were deleted. Also, the heterogeneity of our patient population can be considered to be a limitation of our study, but the study represents a true sample of patients who have an indication for pacing device implantation due to the need for frequent ventricular pacing. Furthermore, the main study was completed in 2014. Since then, there have been technological advances and operator experience in BiV pacing has increased. Finally, a greater number of patients who were initially allocated to BiV stimulation crossed over to the RV group. However, the results of the as-treated analysis revealed no meaningful difference when compared with the intention-to-treat analysis.

In conclusion, superiority of primary implantation of BiV pacing devices compared with RV pacing devices could not be proven in this study, which included patients with high ventricular pacing burden and mostly preserved LV function and normal QRS duration. Instead of a suspected estimated HR of 0.8 or less, the study showed only a point estimator of 0.88 for (i) death or hospitalization due to heart failure and 0.93 for (ii) death alone.

## Supplementary Material

euaf029_Supplementary_Data

## Data Availability

Data are available on request.
