# Correction to: Biventricular vs. right ventricular pacing devices in patients anticipated to require frequent ventricular pacing (BioPace)

**DOI:** 10.1093/europace/euaf253

**Published:** 2025-10-09

This is a correction to: Reinhard C Funck, Hans-Helge Müller, Maurizio Lunati, Luc De Roy, Norbert Klein, Eckhard Meisel, Goran Milasinovic, Mark D Carlson, Michael Wittenberg, Gerhard Hindricks, Jean-Jacques Blanc, on the behalf of the Biventricular Pacing for Atrioventricular Block to Prevent Cardiac Desynchronization (BioPace) Trial Investigators, Biventricular vs. right ventricular pacing devices in patients anticipated to require frequent ventricular pacing (BioPace), *EP Europace*, Volume 27, Issue 3, March 2025, euaf029, https://doi.org/10.1093/europace/euaf029

In the originally published version of the manuscript, there were errores in the graphical abstract. The graphs were duplicated and the upper right box should read: “RV group (n = 908)” instead of: “BiV group (n = 908).”


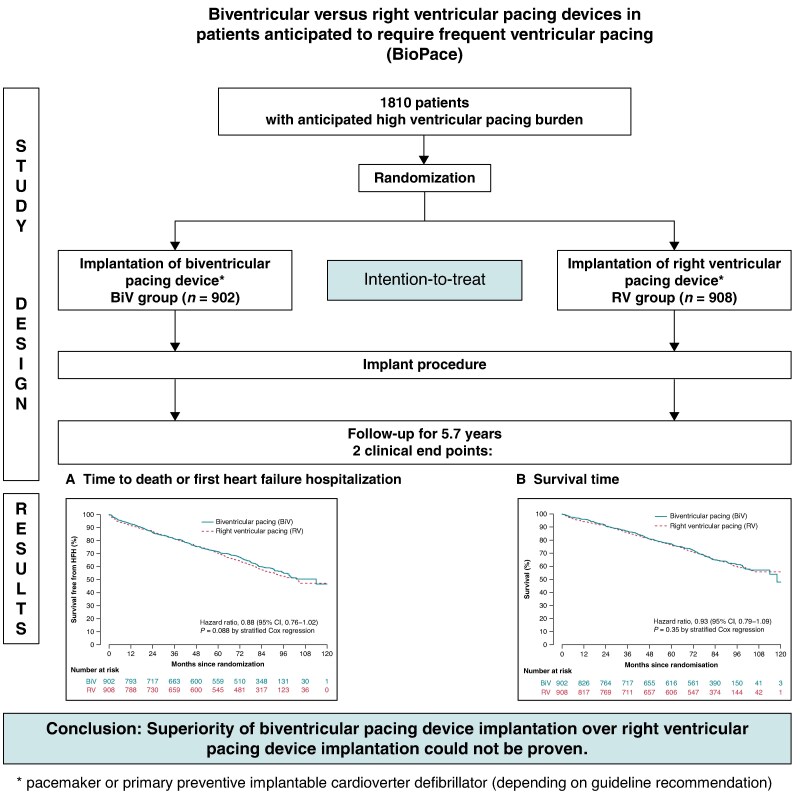


instead of:


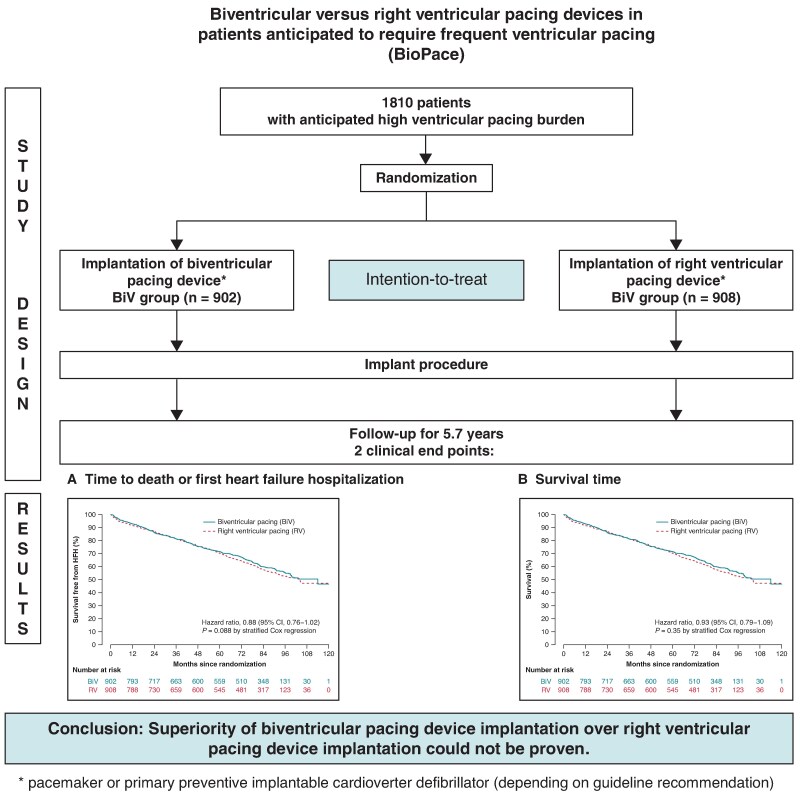


In Figure 2, the graphs in panels A and B were identical. Figure 2B is now emended with the correct graph.


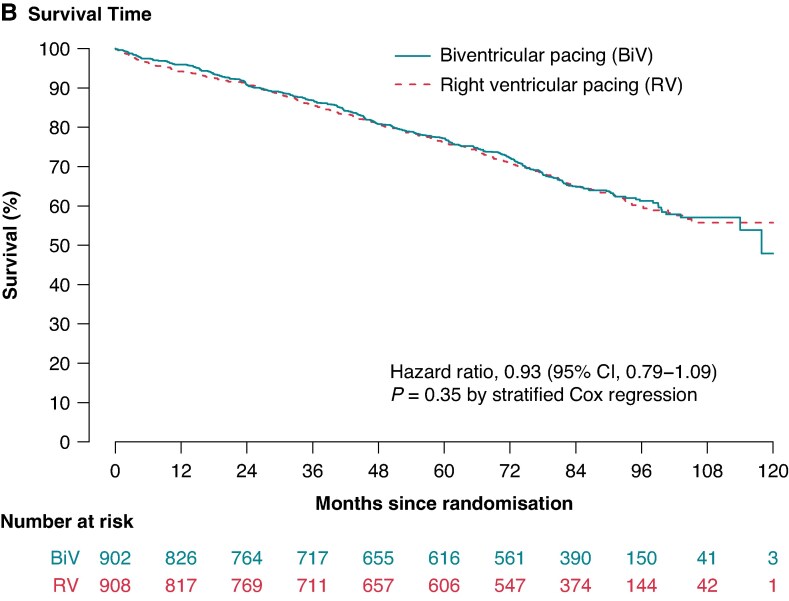


instead of:
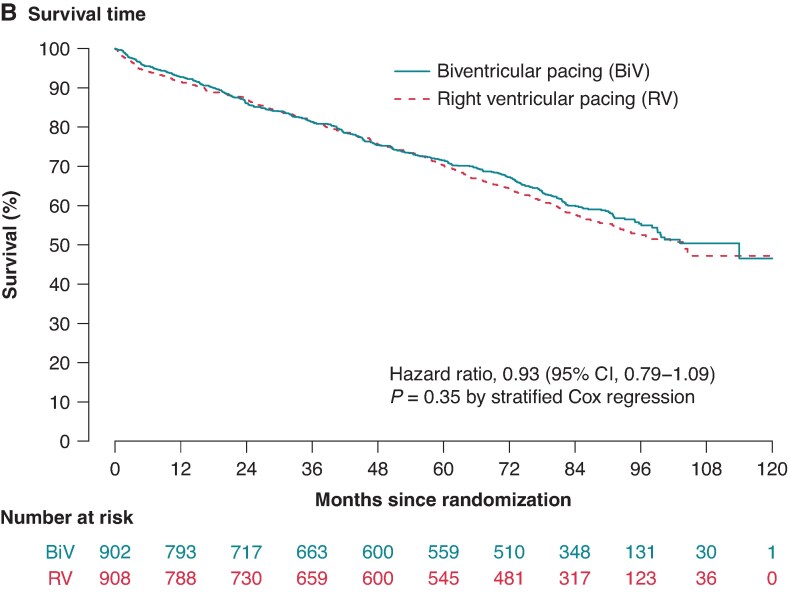


The emendations have been made to the article.

