# Supplementary material for: Biventricular vs. right ventricular pacing devices in patients anticipated to require frequent ventricular pacing (BioPace)
Source: Europace. 2025 Mar 19;27(3):euaf029. doi: 10.1093/europace/euaf029 (PMC11921422; doi:10.1093/europace/euaf029)
Supplement: euaf029_Supplementary_Data [file euaf029_supplementary_data.docx]

**-Online Supplement-**

**Biventricular versus right ventricular pacing devices in patients
anticipated to require frequent ventricular pacing (BioPace)**

Reinhard C. Funck, M.D^1*^, Hans-Helge Müller, PhD.^2*^, Maurizio Lunati, M.D.^3^, Luc De Roy, M.D.^4^, Norbert Klein, M.D.^5^, Alfredo Vicentini, M.D.^6^†, Eckhard Meisel^7^, M.D., Goran Milasinovic, M.D.^8^, Mark Carlson M.D.^9^, Michael Wittenberg, PhD^10^, Gerhard Hindricks, M.D.^11^, Jean-Jacques Blanc, M.D.^12^ for the **B**iventricular Pacing for Atr**i**oventricular Bl**o**ck to **P**revent C**a**rdia**c** D**e**synchronization (BioPace) Trial Investigators**

1. Philipps-Universität Marburg, Klinik für Innere Medizin - Kardiologie, Baldingerstr., 35033 Marburg
2. Philipps-Universität Marburg, Institut für Medizinische Bioinformatik und Biostatistik, Hans-Meerwein-Str. 6, 35043 Marburg, Germany
3. Ospedale Niguarda, Cardiologia 3 – Elettrofisiologia, Piazza Ospedale Maggiore 3, 20162 Milano, Italy
4. CHU Namur, Cardiology Department, Ave G. Therasse 1-8, 5530 Yvoir, Belgium
5. Klinikum St. Georg gGmbH, Department of Cardiology, Delitzscher Str. 141, 04129 Leipzig, Germany
6. Casa di Cura Polispecialistica Dott. Pederzoli, Cardiologia, 37019 Peschiera del Garda (Verona), Italy, †Died April 15, 2022
7. Praxisklinik HerzKreislauf am WASA-Platz, August-Bebel-Str. 33, 01219 Dresden, Germany
8. Klinicki centar Srbije, Pacemaker Clinic, 11000 Beograd, Serbia
9. Abbott, Cardiac Rhythm Management, 15900 Valley View Court, Sylmar, CA, USA 91
10. Coordinating Center for Clinical Trials of the Philipps-University of Marburg, Karl-von-Frisch-Str. 4, 35043 Marburg, Germany
11. Campus Charité Mitte, Charitéplatz 1, 10117 Berlin, Germany
12. Brest University Hospital, Boulevard Tanguy Prigent, 29609 Brest, France

Contents

[1 Investigators and Collaborators 5](#_Toc177648935)

[2 Committees 8](#_Toc177648936)

[2.1 Members of the Steering Committee 8](#_Toc177648937)

[2.2 Members of the Independent Event Adjudication Committee 8](#_Toc177648938)

[2.3 Members of the Data Safety Monitoring Board 8](#_Toc177648939)

[3 Summary of amendments to the study protocol 9](#_Toc177648940)

[3.1 Amendment No. 1 (21JAN2004) 9](#_Toc177648941)

[3.1.1 Changes 9](#_Toc177648942)

[3.1.2 Changes in SAP: 9](#_Toc177648943)

[3.1.3 Rationale 9](#_Toc177648944)

[3.1.4 Reference List 10](#_Toc177648945)

[3.2 Amendment No. 2 (30JUN2004) 10](#_Toc177648946)

[3.2.1 Changes 10](#_Toc177648947)

[3.2.2 Changes in SAP: 10](#_Toc177648948)

[3.2.3 Rationale 10](#_Toc177648949)

[3.3 Amendment No. 3 (22JUL2004) 10](#_Toc177648950)

[3.3.1 Changes 10](#_Toc177648951)

[3.3.2 Changes in SAP: 10](#_Toc177648952)

[3.3.3 Rationale 10](#_Toc177648953)

[3.4 Amendment No. 4 (20DEC2004) 10](#_Toc177648954)

[3.4.1 Changes 10](#_Toc177648955)

[3.4.2 Changes in SAP: 10](#_Toc177648956)

[3.4.3 Rationale 11](#_Toc177648957)

[3.5 Amendment No. 5 (07JUL2005) 11](#_Toc177648958)

[3.5.1 Changes 11](#_Toc177648959)

[3.5.2 Changes in SAP 11](#_Toc177648960)

[3.5.3 Rationale 11](#_Toc177648961)

[3.5.4 Reference List 12](#_Toc177648962)

[3.6 Amendment No. 6 (08JAN2007) 13](#_Toc177648963)

[3.6.1 Changes 13](#_Toc177648964)

[3.6.2 Changes in SAP 13](#_Toc177648965)

[3.6.3 Rationale 13](#_Toc177648966)

[3.7 Amendment No. 7 (21MAR2014) 14](#_Toc177648967)

[3.7.1 Changes 14](#_Toc177648968)

[3.7.2 Changes in SAP 14](#_Toc177648969)

[3.7.3 Rationale 14](#_Toc177648970)

[3.7.4 Reference List 15](#_Toc177648971)

[4 Patient Screening 15](#_Toc177648972)

[5 Implanted Devices 15](#_Toc177648973)

[5.1 Implantable Pulse generators 15](#_Toc177648974)

[5.2 Implantable cardioverter defibrillators 16](#_Toc177648975)

[5.3 Lead system 16](#_Toc177648976)

[6 Atrioventricular synchronization 16](#_Toc177648977)

[6.1 Reference List 16](#_Toc177648978)

[7 Randomization Algorithm 16](#_Toc177648979)

[8 Additional Results 17](#_Toc177648980)

[8.1 Trial Profile 17](#_Toc177648981)

[8.2 Results in the *intention-to-treat* cohort 19](#_Toc177648982)

[8.3 Results in the cohort with implantation of study devices 20](#_Toc177648983)

[8.4 Results on functional status at 3 months after initial implantation 21](#_Toc177648984)

[8.5 Sensitivity Analyses 22](#_Toc177648985)

[8.6 Safety issues 22](#_Toc177648986)

[9 Figures and Tables 23](#_Toc177648987)

List of [Supplementary Figures and Supplementary Tables](#_Toc490120921)

| Figure S1: Time to death or hospitalization due to heart failure (Forest plot, subgroups) .…............................ | 23 |
| --- | --- |
| Figure S2: Survival Time (Forest plot, subgroups) .………………………………………………………………........................... | 24 |
| Figure S3: Time to cardiovascular death or hospitalization due to heart failure (Kaplan-Meier estimate) ....... | 25 |
| Figure S4: Time to cardiovascular death or hospitalization due to heart failure (Forest plot, subgroups) ....... | 26 |
| Figure S5: Time to cardiovascular death (Kaplan-Meier estimate) ..……………………………………........................... | 27 |
| Figure S6: Time to cardiovascular death (Forest plot, subgroups) ……….……………………………….......................... | 28 |
| Figure S7: Time to heart failure manifestation resulting in death or hospitalization (Kaplan-Meier estimate) | 29 |
| Figure S8: Time to heart failure manifestation resulting in death or hospitalization (Forest plot, subgroups) . | 30 |
| Figure S9: Median ventricular pacing percentages in both study arms during the study ……............................ | 31 |
| Table S1: Median ventricular pacing percentages in both study arms during the study ……….......................... | 32 |
| Figure S10: Median ventricular pacing percentages in both study arms during the study  for LVEF ≤ 35% …..........................…………………………………………………………………..…………………………… | 33 |
| Table S2: Median ventricular pacing percentages in both study arms during the study  for LVEF ≤ 35% ………………………………………………………………………….…………………………………………………… | 34 |
| Figure S11: Median ventricular pacing percentages in both study arms during the study  for LVEF 36% to 50% …………………………………………………………....…………………………………….……………… | 35 |
| Table S3: Median ventricular pacing percentages in both study arms during the study  for LVEF 36% to 50% …………………………………………………………………………………………………………..…………. | 36 |
| Figure S12: Median ventricular pacing percentages in both study arms during the study  for LVEF > 50% ……………………………………………………………….……………………………………………..……………. | 37 |
| Table S4: Median ventricular pacing percentages in both study arms during the study  for LVEF > 50% ………………………………………………………………………….………………………………….…………….… | 38 |
| Table S5: Functioning of LV lead at three months after initial implantation …………………….……………………….. | 39 |
| Table S6: Number of patients with crossover from BiV to RV pacing and number  of patients with crossover from RV to BiV pacing by time of crossover   later than three months after initial implantation ……………………………………………….………………………... | 40 |
| Table S7: Serious adverse events – number of events ………………………………………………………….………………………. | 41 |
| Table S8: Non serious adverse events – number of events …………………………………………….…………………………….. | 42 |
| Table S9: Serious adverse events – number of patients ………………………………………………………………………………… | 43 |
| Table S10: Non serious adverse events – number of patients …………………………………………….…………………………. | 44 |

# Investigators and Collaborators

We would like to acknowledge the support from the following recruiting sites and investigators of the BioPace study in accomplishing the study:

**Australia**

Voltaire Jose M. Nadurata, Dr., Bendigo Health Care Group, Bendigo VIC, Australia

**Austria**

Georg Grimm, Prim. Univ.-Prof. Dr., Landeskrankenhaus Klagenfurt, Klagenfurt, Austria

Kurt Huber, Prim. Univ.-Prof. Dr., Wilhelminenspital der Stadt Wien, Wien, Austria

Peter Siostrzonek, Prim. Univ.-Prof. Dr., Krankenhaus der Barmherzigen Schwestern Linz, Linz a.d. Donau, Austria

**Belgium**

Dominique Blommaert, Prof., CHU UCL Namur, Yvoir, Belgium

Marc Castadot, Dr., Cliniques St. Jean, Brussels, Belgium

Richard Deperon, Dr., Clinique Notre Dame de Grâce, Gosselies, Belgium

Georges Henri Mairesse, Dr., Cliniques du Sud-Luxembourg, Arlon, Belgium

Eric Stoupel, Prof. Dr., Hôpital Universitaire Erasme, Brussels, Belgium

Yves Vandekerckhove, Dr., AZ - St. Jan, Brugge, Belgium

Chris Vrints, Prof. Dr., UZA, Edegem, Belgium

Djamel Zenagui, Dr., Hopitaux Iris, Bracops, Belgium

**Canada**

Felix Paredes, Dr., Centre Hospitalier Universitaire de Sherbrooke - Hopital Fleurimont, Sherbrooke Quebec, Canada

John Parker, Dr., Mount Sinai Hospital, Toronto ON, Canada

**Estonia**

Rein Kolk, Dr., Tartu University Hospital, Tartu, Estonia

**France**

Frédéric Anselme, Prof., CHU Charles Nicolle, Rouen, France

Jean-Marc Davy, Prof., Hôpital Arnaud de Villeneuve, Montpellier, France

Jean-Claude Deharo, Prof., Groupe Hospitalier de la Timone, Marseille, France

Stéphane Dennetiere, Dr., Centre Hospitalier Victor Provo, Roubaix, France

Jean-Marc Dupuis, Dr., CHRU, Angers, France

Yves Ètienne Dr., CHRU La Cavale Blanche, Brest, France

Pierre Graux, Prof., Hôpital Saint Philibert, Lomme, France

Gaël Jauvert, Dr., Clinique Bizet, Paris, France

Salem Kacet, Prof., CHRU de Lille, Lille, France

Jean-Ernest Poulard, Dr., Service de Cardiologie, Abbeville, France

Jean-Luc Rey, Prof., Service de Cardiologie, Amiens, France

**Germany**

Christoph Axthelm, Dr. med., Klinikum Pirna, Pirna, Germany

Juergen Brömsen, Dr. med., Stiftsklinik Augustinum, München, Germany

Matthias Czech, Dr. med., Asklepios-ASB Klinik Radeberg, Radeberg, Germany

Wilfried Dänschel, Dr. med., MVZ am Küchwald GmbH, Chemnitz, Germany

Claudia Daub, Dr. med., Elisabeth-Krankenhaus, Recklinghausen, Germany

Johann C. Geller, Prof. Dr. med., Zentralklinik Bad Berka GmbH, Bad Berka, Germany

Rainer Grove Dr. med., Schüchtermannklinik, Bad Rothenfelde, Germany

Kerstin Hahlweg, Dr. med., Klinikum Barnim GmbH Werner-Forsmann-Krankenhaus, Eberswalde, Germany

Christopher Piorkowski, PD Dr. med., Herzzentrum Leipzig GmbH, Leipzig, Germany

Thomas Ketteler, Dr. med., Helios Klinikum Aue GmbH, Aue, Germany

Susanne Koelsch, Dr., Klinikum der Philipps-Universität Marburg, Marburg, Germany

Bernd Lemke, Prof. Dr. med., Klinikum Lüdenscheid, Lüdenscheid, Germany

Michael Liebetrau PD Dr. med., HELIOS Klinik Blankenhain, Blankenhain, Germany

Stephan Albrecht, Dr. med., Praxisklinik Dresden, Germany

Gerhard Oltmanns, PD Dr. med., DRK Krankenhaus Sömmerda GmbH, Sömmerda, Germany

Christian Perings, Prof. Dr. med., St.-Marien-Hospital, Lünen, Germany

Dietrich Pfeiffer, Prof. Dr. med., Universität Leipzig, Leipzig, Germany

Karsten Reinig, Dr. med., Helios Klinik Gotha/Ohrdruf, Gotha, Germany

Norman Rueb, Dr. med., Kliniken Ludwigsburg-Bietigheim gGmbH, Ludwigsburg, Germany

Georg Sabin Prof., Dr. med., Elisabeth-Krankenhaus, Essen, Germany

K. J. G. Schmailzl, Prof. Dr. Dr. med., Ruppiner Kliniken, Neuruppin, Germany

Peter Sick PD, Dr. med., Krankenhaus Barmherzige Brüder, Regensburg, Germany

Horst Sievert, Prof. Dr. med., Cardiovasculaeres Zentrum, Frankfurt am Main, Germany

Joern Schmitt, PD Dr. med., Klinikum der Justus-Liebig-Universität, Giessen, Germany

Stefan Steiner, Dr. med., Herz- und Kreislaufzentrum Rotenburg an der Fulda, Rotenburg an der Fulda, Germany

Istvan Szendey, Dr. med., Kliniken Maria Hilf GmbH - Krankenhaus St. Franziskus, Mönchengladbach, Germany

Peter Weissmüller, Prof. Dr. med., Allgemeines KRH Hagen, Hagen, Germany

Peter Weitkamp, Dr. med., Klinikum Kreis Herford, Herford, Germany

Ralf Zahn, Prof. Dr. med., Klinikum der Stadt Ludwigshafen, Ludwigshafen, Germany

**Italy**

Giovanni Luca Botto, Dr., Ospedale S. Anna, Como, Italy

Di Girolamo Enrico, Dr., Osp. Clinicizzato S.S. Annunziata, Chieti, Italy

Luigi Leonzio, Dr., Ospedale Renzetti, Lanciano, Italy

Giovanni Luzzi, Dr., Policlinico consorziale di Bari, Bari, Italy

Ederina Mulargia, Dr., Ospedale Niguarda Ca'Granda, Milano, Italy

Luigi Padeletti, Prof., Az.Ospedaliera-Universitaria Careggi Clinica Medica, Firenze, Italy

Giovanni Carreras, Dr., Azienda Ospedaliera S.Maria, Terni, Italy

Francesco Solimene, Dr., Casa Di Cura Montevergine, Mercogliano, Italy

Tiziano Toselli, Dr., Azienda Ospedaliera Universitaria S. Anna, Ferrara, Italy

Diego Vaccari, Dr., Ospedale Carretta, Montebelluna, Italy

Roberto Verlato, Dr., Ospedale di Camposampiero, Camposampiero, Italy

Antonio Fusco, Dr., Casa Di Cura Polispecialistica Dott. Pederzoli, Peschiera del Garda (Verona), Italy

**Netherlands**

A.M.W. Alings, Dr., Dr., Amphia Ziekenhuis, Breda, Netherlands

B. Dijkman, Dr., Maasstadziekenhuis, Rotterdam, Netherlands

S.A.M. Saïd, Dr., Streekziekenhuis Midden-Twente, Hengelo, Netherlands

W.G. de Voogt, Dr., St. Lucas Andreas Ziekenhuis, Amsterdam, Netherlands

J.W.M.G. Widdershoven, Dr., Twee Steden Ziekenhuis, Tilburg, Netherlands

**Norway**

Finn Tore Gjestvang, Dr., Sørlandets Sykehus HF Kristiansand, Kristiansand, Norway

Torstein Hole, Dr., Helse Sunnmøre HF Ålesund Sjukehus, Ålesund, Norway

Knut Tore Lappegard, Dr., Nordlandssykehuset HF Bodø, Bodø, Norway

Dennis Nilsen, Dr., Sentralsykehuset I Rogaland - Helse Vest, Stavanger, Norway

**Poland**

Jacek Wilczek, Dr., Samodzielny Publiczny Szpital Kliniczny N° 7 Ślaskiej Akademii Medycznej w Katowicach Górnoślaskie, Centrum Medyczne, Katowice, Poland

Jerzy Spikowski, Dr., Wojewódzki Szpital Specjalistyczny we Wrocławiu, WrocŁaw, Poland

**Serbia**

Lazar Angelkov, Dr, Institute for cardiovascular disease – Dedinje, Belgrade, Serbia

Velibor Jovanović, Dr, Clinical Center of Serbia - Pacemaker Center, Belgrade, Serbia

Zoran Perisic, Prof. Dr, Clinical center of Nis, Nis, Serbia

Vasilije Topalov, Prof. Dr, Institute for cardiovascular desease of Vojvodina, Sremska Kamenica, Serbia

**Sweden**

Thomas Aronsson, Dr., Centrallasarettet i Växjö, Växjó, Sweden

Viveka Frykman, Ö.L, Danderyds Sjukhus AB, Medicinska Kliniken, Danderyd, Sweden

Fredrik Gadler, Dr., Karolinska Universitetssjukhuset-Solna, Stockholm, Sweden

Björn Kjellman, Dr., Karolinska Institutet, Stockholm Sodersjukhuset, Sweden

**Tunisia**

Salem Kachboura, Prof., CHU Abderrahmane Mami, Ariana, Tunisia

**United Kingdom**

Craig S Barr, Dr., Russell’s Hall Hospital, Dudley, United Kingdom

Ian Beeton, Dr., St Peter’s Hospital, Chertsey, United Kingdom

Guy Haywood, Dr., Derriford Hospital, Plymouth, United Kingdom

Jonathan Panting, Dr., Good Hope Hospital, Sutton Coldfield, United Kingdom

David Justin Wright, Dr., Liverpool Heart and Chest Hospital, Liverpool, United Kingdom

Zaheer Yousef, Dr., University Hospital of Wales, Cardiff, United Kingdom

**Collaborators**

We would also like to acknowledge the support from the Center for Clinical Trials of the Philipps-University Marburg (KKS Marburg) for providing randomization (Maik Hahmann), central data management and analysis (Anika Venter) as well as project management for Germany (Carmen Schade-Brittinger).

# Committees

## Members of the Steering Committee

Prof. Jean-Jacques Blanc, Brest, France

Dr. Reinhard C. Funck, Marburg and Bad Hersfeld, Germany

Pr. Maurizio Lunati, Milano, Italy

Prof. Luc De Roy, Yvoir, Belgium

Prof. Gerhard Hindricks, Leipzig and Berlin, Germany

Dr. Vince Paul, Perth, Australia

We acknowledge the support from the members of the Independent Event Adjudication Committee and the Data Safety Monitoring Board.

## Members of the Independent Event Adjudication Committee

Prof. Jean-Claude Daubert, Rennes, France (chair)

Prof. Luigi Tavazzi, Cotignola, Italy

Prof. Kristian Thygesen, Aarhus, Denmark

## Members of the Data Safety Monitoring Board

Prof. Cecilia Linde, Stockholm, Sweden (chair)

Prof. Christophe Leclercq, Rennes, France

Prof. Hans J. Trampisch, Bochum, Germany (statistician)

# Summary of amendments to the study protocol

In the following the respective changes in the study protocol, the changes in the statistical analysis plan (SAP), the rationale for both and, as far as necessary the respective references are listed.

## Amendment No. 1 (21JAN2004)

### Changes

Inclusion Criteria were modified:

- LVEDD/height ≥ 30 mm/m criterion was removed
- LVEF ≤ 45% criterion was removed
- Patients scheduled for an AV-node ablation were added

RV lead placement in the RVOT was tolerated

### Changes in SAP:

The Statistical Analysis Plan (SAP) implemented:

- Stratification regarding LVEF for randomization and adjustment in primary outcome analysis was according to the extension of the inclusion criteria extended to the 3 strata LVEF ≤ 35%, 35% < LVEF ≤ 50%, and LVEF > 50%. Statistical redesign was postponed.
- A sensitivity analysis of the primary outcome which additionally adjusts for gender and age at randomization was included.

### Rationale

On January 21, 2004 the members of the BioPace Study Board decided to modify the inclusion criteria of the study. It was decided to make the study accessible not only for patients with dilated left ventricles and reduced left ventricular ejection fraction (≤ 45 %) but also for patients with normal ventricles. This decision was taken in part to enhance patient recruitment. The incidence of patients needing ventricular pacing and left ventricular dysfunction prior to device implantation had been overestimated. The study steering committee hypothesized that LVEF deterioration during ventricular pacing in patients with initially normal LVEF could be prevented by biventricular pacing. Hence, the steering committee decided to also include patients with preserved left ventricular ejection fraction and removed the following inclusion criteria:

- LVEDD/height ≥ 30 mm/m
- LVEF ≤ 45 %

The decision was based on available data on left ventricular performance during right ventricular pacing. In 1997, left ventricular performance in structurally normal hearts in infants was found to be impaired by right ventricular apical pacing^1^. This is in accordance with more recent data suggesting that the potential deleterious effects of right ventricular pacing may not be confined to patients with left ventricular dilatation and dysfunction^2^. In that study, hospitalization rates and atrial arrhythmic burden appeared to be substantially increased by the desynchronizing effect of right ventricular pacing. Based on these and other data and since patient hospitalization and incidence of atrial fibrillation were secondary endpoints of the BioPace study, the steering committee decided to expand the study to all patients with the mentioned indications. In this situation in which it was difficult to estimate the effects of right ventricular compared to biventricular pacing in the different subgroups we took advantage of the adaptive group sequential design approach^3^. When including patients with normal or nearly normal left ventricles it was anticipated that a later amendment would be needed to increase the follow-up duration, the number of patient enrollments and the number of events to be observed.

Independently, another consequence of including patients with normal or nearly normal left ventricular structure and function was that more heterogeneity in prognosis had to be expected. Concerning the LVEF criterion in the primary stratified statistical analysis, the group of patients with LVEF > 35% was expected to become the predominant subgroup. Accounting for possible heterogeneity in the statistical analysis, it was planned to split up this group in patients with borderline left ventricular function (35% < LVEF ≤ 50%) and patients with nearly normal to normal left ventricular systolic function with LVEF > 50%^4^.

### Reference List

1. Karpawich PP, Mital S. Comparative left ventricular function following atrial, septal, and apical single chamber heart pacing in the young. Pacing Clin Electrophysiol 1997; 20: 1983-1988.

2. Sweeney MO, Hellkamp AS, Ellenbogen KA et al. Adverse effect of ventricular pacing on heart failure and atrial fibrillation among patients with normal baseline QRS duration in a clinical trial of pacemaker therapy for sinus node dysfunction. Circulation 2003; 107: 2932-2937.

3. Müller HH, Schäfer H. Adaptive group sequential designs for clinical trials: Combining the advantages of adaptive and of classical group sequential approaches. Biometrics 2001; 57: 886-891.

4. McMurray JJV, Stewart S. The burden of heart failure. EHJ Supplements 2003; 5: I3-I13.

## Amendment No. 2 (30JUN2004)

### Changes

Up to 30 patients in whom Frontier II devices had been implanted were followed until 3-months post-implant. This amendment was applicable only in a selected number of centers.

### Changes in SAP:

This did not have any implications for the SAP of the BioPace study.

### Rationale

The New Technology Assessment for the Frontier II device was combined with the study.

## Amendment No. 3 (22JUL2004)

### Changes

Up to 30 patients in whom Quicksite T leads were implanted were followed until 3-months post-implant. This amendment was applicable only in a selected number of centers.

### Changes in SAP:

This did not have any implications for the SAP of the BioPace study.

### Rationale

The New Technology Assessment for the Quicksite T lead device was combined with the study.

## Amendment No. 4 (20DEC2004)

### Changes

The recruiting period was prolonged from 18 to 36 months.

### Changes in SAP:

The prolongation of the recruitment period from 18 to 36 months was incorporated into the SAP.

### Rationale

In the initial BioPace study protocol a recruiting period of 18 months was assumed. Because of the dragging start-up-phase (see also rationale of amendment No. 1) the study board decided to prolong the recruiting period to a minimum of 36 months.

## Amendment No. 5 (07JUL2005)

### Changes

- Implantable Cardioverter Defibrillator (ICD) patients with primary preventive ICD indications were allowed to be recruited.
- Any commercially available ICD lead could be used.
- Frontier II device was added to the devices that could be used.
- The Case Report Forms (CRFs) were redesigned.
- The Echo-protocol was simplified.
- The Lab tests were removed from the CRFs.

### Changes in SAP

Stratification regarding LVEF considered for randomization and adjustment in primary outcome analysis was enlarged to the 4 strata LVEF ≤ 35% without ICD, LVEF ≤ 35% with ICD, 35% < LVEF ≤ 50%, and LVEF > 50% according to the heterogeneity induced when allowing inclusion of patients with an indication for an ICD.

### Rationale

One of the major determinants of patient mortality is the degree of left ventricular dysfunction. At the time of amendment 5 it had become widely accepted that patients with ejection fractions ≤ 35% represent a high-risk population for sudden cardiac death, even if prior arrhythmic events are lacking. Two major international prospective randomized trials on primary prevention of sudden cardiac death were the Multicenter Automatic Defibrillator Implantation Trial (MADIT II)^1^ and the Sudden Cardiac Death in Heart Failure Trial (SCDHeFT)^2^.

**MADIT II** investigated the effect of implantable cardioverter defibrillator (ICD) therapy on sudden cardiac death reduction in patients with history of myocardial infarction and left ventricular ejection fractions (LVEF) ≤ 30%. In the MADIT II study LVEF was 23.5% in the ICD-arm and 23.6% in the conventional arm and most of the patients were in NYHA class I – III. After a follow-up period of 20 months overall mortality was 14.2% in the ICD arm and 19.8% in the conventional arm. The ICD reduced the risk for sudden cardiac death by 28%.

The **SCD-HeFT** study included heart failure patients (NYHA class II-III) with ischemic and non-ischemic heart disease and slightly less impaired left ventricular function (ejection fraction ≤ 35%). Mean left ventricular ejection fraction was 25% in the study. Spontaneous overall annual mortality was predicted to equal 10% in the placebo-arm of the SCD-HeFT study but the final result was 7.2% per year over a period of 5 years. Overall mortality at the end of the study was 29% in the placebo group, 28% in the amiodarone group and 22% in the ICD group. The relative risk reduction achieved by the ICD was 23% compared to placebo therapy. These data showed that defibrillators can reduce the risk for sudden cardiac death in patients with chronic heart failure and high risk for sudden cardiac death which was mainly defined by the degree of left ventricular dysfunction. Hence, the BioPace study design was adapted to include patients who fulfilled the inclusion criteria and who met the MADIT II and/or SCD-HeFT LVEF inclusion criteria. Thus, ICD implant for primary sudden cardiac death prevention was no longer an exclusion criterion for the BioPace study as long as at least one of the pacing indications listed under “Inclusion Criteria” of the BioPace study protocol was fulfilled. This amendment to the BioPace study was therefore a logic reaction of the steering committee to recently published data on primary prevention of sudden cardiac death and to recent guideline modifications^3^.

This amendment did not alter the inclusion criteria of the study. It merely modified the exclusion criteria in the way that ICDs were allowed to be used as pacing devices in patients with severely reduced left ventricular ejection fraction for primary prevention of sudden cardiac death due to lethal ventricular tachyarrhythmias in addition to the correction of bradycardia. The decision to implant an ICD instead of a pacemaker was only based on the insight that patients with severely impaired left ventricular ejection fractions are prone to sudden cardiac death. No other accepted tachyarrhythmia indication for the implantation of an ICD was allowed to be present.

### Reference List

1. Moss AJ, Zareba W, Hall WJ et al. Prophylactic implantation of a defibrillator in patients with myocardial infarction and reduced ejection fraction. N Engl J Med. 2002;346:877-883.

2. Bardy GH, Lee KL, Mark DB et al. Amiodarone or an implantable cardioverter defibrillator for congestive heart failure. N Engl J Med. 2005;352:225-237.

3. Gregoratos G, Abrams J, Epstein AE et al. ACC/AHA/NASPE 2002 guideline update for implantation of cardiac pacemakers and antiarrhythmia devices: summary article. A report of the American College of Cardiology/American Heart Association Task Force on Practice Guidelines (ACC/AHA/NASPE Committee to Update the 1998 Pacemaker Guidelines). J Cardiovasc Electrophysiol. 2002; 13:1183-1199.

## Amendment No. 6 (08JAN2007)

### Changes

- The sample size was extended to 1800 patients
- Survival time was defined as the only primary endpoint.
- Quality of Life questionnaire, 6MWT & Echo are only required until 24 months post-implant.
- The follow-up schedule was modified after 24 months follow-up.
- The inclusion period was prolonged.

### Changes in SAP

The statistical analysis was re-planned as a consequence of the extension of inclusion criteria, particularly considering Amendment No. 1 which allowed patients with LVEF > 45% to be included.

- Only survival time was left as a primary endpoint, regardless what the reason for death was. This was in contrast to the former definition in which deaths where any influence from the cardiac disease could be excluded were censored.
- The assumptions on median survival times in the treatment groups were re-specified to 8 years in the RV group and to 10 years in the BiV group.
- The number of events (deaths) to be observed at the final analysis was increased to 635.
- The sample size was extended to 1800 patients assuming that, due to drop-outs, up to 15% of survival time could be lost to follow-up.
- The inclusion period was prolonged.
- The prognostic relevance of gender and age was recognized. The primary Cox regression model was set up to adjust also for gender and age, instead of only to be used in a sensitivity analysis.
- Functional capacity (6 min walk test) and quality of life (Minnesota^©^ questionnaire) were changed from primary to secondary outcomes until 24 months post-implant.
- Time to cardiovascular death was included as key secondary endpoint for explanatory purpose.
- Evaluation of safety criteria of the implantation of a biventricular pacemaker system became a secondary objective.

### Rationale

The BioPace DSMB reviewed the study data available in December 2006 and recommended to follow the statistician´s proposal which implied an extension of the sample size from 1200 to 1800 patients as well as a modification of the follow-up period according to the study endpoint. It was determined that the primary study endpoint would be limited to survival time and the number of deaths to be observed would be increased from 382 to 635. In addition, it was determined that time to cardiovascular death would be the first secondary endpoint and functional capacity as measured by the distance covered in the 6-minute walk test and Health related Quality of Life measured by the Minnesota Living With Heart Failure questionnaire^©^, both evaluated at 12 and 24 months after implantation, were changed to secondary endpoints.

These changes were made because the inclusion of patients with normal LVEF was expected to make the 6MWT and the MLWHFQ inappropriate.

## Amendment No. 7 (21MAR2014)

### Changes

The composite endpoint consisting of overall survival time and time to first hospitalization due to heart failure was added.

### Changes in SAP

The statistical analysis was re-designed in order to incorporate the composite endpoint, finally ending up with two co-primary endpoints.

- The co-primary composite endpoint was integrated as confirmatory endpoint in the SAP by performing hierarchical statistical testing. It was decided to first test the composite endpoint at 5% two-sided significance level after 635 events were expected to be observed for the composite endpoint.
  In case of statistical significance of this first test, it was decided to statistically test the survival time endpoint at 5% two-sided significance level after 635 deaths were expected to be observed. For the scenario that the first test failed to confirm superiority of biventricular pacing compared to right ventricular pacing, it was decided to test descriptively survival time based on the survival information observed until the time when the co-primary endpoint was tested.
- Time to first hospitalization due to heart failure or cardiovascular death was included as a key secondary composite endpoint for explanatory purpose.
- Handling of missing data was specified for the primary endpoint analysis.

### Rationale

Considering the high survival rate of the patients recruited particularly following the implementation of amendment 1, the following questions arose:

1. Is it useful to continue the trial?
2. Might the detection of a clinically important difference possibly be missed despite the size and duration of the trial if survival time was kept as primary endpoint?
3. Is it useful to incorporate the composite endpoint survival time or time to first hospitalization due to heart failure as another primary endpoint, especially to evaluate the concept of biventricular pacing in this type of patients?

In order to answer these questions, a grouped analysis was performed by the statistician in August 2012. This interim analysis was performed on the basis of 432 deaths that had been observed until so far (68% of the number of events planned in amendment 6). For this purpose the allocation of the data to individual patients was actively blinded by the trial data management before they were handed over to the statistician. The results were presented only to the members of the DSMB and neither to the steering committee nor to the sponsor. On the basis of the results of this interim analysis the DSMB recommended to continue the study. No further recommendations, whether to add or not to add a specific co-primary endpoint, were made by the DSMB.

Given the facts that survival time had been defined as the only endpoint of the study (Amendment 6) and that this endpoint had already been chosen when LVEF as inclusion criterion was restricted to ≤ 45% there were considerations that a clinically relevant difference between the two study arms could be missed despite the size and the duration of the study. Hence, the question arose either to replace the endpoint survival time or to add a co-primary endpoint. Despite the fact that – according to the conditional rejection probability principle^1,2^ – there would have been the option to skip the statistical analysis of survival time (Amendment 6) the steering committee and the sponsor decided to follow the recommendation of the statistician not to use this option. Therefore, neither the endpoint survival time was abolished as a primary endpoint nor were the statistical test or the planned number of observed deaths changed. Instead, the steering committee and the sponsor decided to add a composite endpoint consisting of survival time and time to first hospitalization due to heart failure, a well-established endpoint in heart failure trials^3-7^ and particularly in biventricular pacing trials. The DSMB accepted the incorporation of this composite endpoint. This endpoint was chosen because it was considered to be

1. an important heart-failure orientated and hence meaningful endpoint
2. more sensitive than all-cause mortality in order not to leave a difference between RV and BiV pacing falsely undetected and
3. more specific than survival time.

The survival time endpoint was maintained as co-primary endpoint, primarily because it has the advantage of being patient orientated, easy to measure, and because it is resistant to patient or observer bias.

The period from August 2012 to March 2014 was needed to establish and to test all preconditions so that all reasons for hospitalization could correctly be adjudicated in order to get amendment 7 approved.

### Reference List

1. Müller HH, Schäfer H. Adaptive group sequential designs for clinical trials: combining the advantages of adaptive and of classical group sequential approaches. Biometrics 2001;57:886-91.

2. Müller HH, Schäfer H. A general statistical principle for changing a design any time during the course of a trial. Stat Med 2004;23:2497-508.

3. Higgins SL and al. Cardiac resynchronization therapy for the treatment of heart failure in patients with intraventricular conduction delay and malignant ventricular tachyarrhythmias. J Am Coll Cardiol. 2003 Oct 15

4. Linde C and al. Randomized trial of cardiac resynchronization in mildly symptomatic heart failure patients and in asymptomatic patients with left ventricular dysfunction and previous heart failure symptoms. J Am Coll Cardiol. 2008 Dec 2

5. Moss AJ and al. Cardiac-resynchronization therapy for the prevention of heart failure events. N Engl J Med. 2009 Oct 1

6. Tang AS and al. Cardiac-resynchronization therapy for mild-to-moderate heart failure. N Engl J Med. 2010 Dec 16

7. Curtis AB and al. Biventricular Pacing for Atrioventricular Block and Systolic Dysfunction. N Engl J Med 2013

# Patient Screening

A screening log was initially part of the protocol. Considering the emergency situation of many patients with critical bradycardia the steering committee decided after detailed consideration of the topic that a consequent implementation of a screening log in all study centers and for all patients would not be feasible, mainly because all screened patients would have needed to give their informed consent for being listed in the screening log.

# Implanted Devices

## Implantable Pulse generators

Patients assigned to biventricular pacing received the Model 5510 Frontier 3 _ 2 (St Jude Medical Inc., Sylmar, CA, USA) triple-chamber pacemaker or newer models. Depending on whether sinus rhythm or chronic AF is present, patients randomized to the RV pacing group received conventional single or dual-chamber pulse generators manufactured by St Jude Medical Inc.

## Implantable cardioverter defibrillators

The Epic HF (Model V-339) or any St Jude Medical CRT-D device with comparable study related functions were used in patients randomized to biventricular pacing who required an ICD. No atrial lead was implanted in patients with permanent AF in order to avoid procedural differences compared with the control group.

Depending on the presence or absence of sinus rhythm, the Epic DR (Model V-233) or the Epic þ VR (Model V-196) or any St Jude Medical ICD with comparable study related functions were used in patients randomized to RV pacing who required an ICD. All ICDs were programmed as ‘shock-only devices’ so that shocks were delivered for ventricular fibrillation and fast, hemodynamically not tolerated ventricular tachycardias. The use of antitachycardia pacing therapies was not allowed. This was crucial for data analysis in which every appropriate shock was counted as an event of death.

## Lead system

The Model 1055 K Aescula or Model 1056 K QuickSite Left Heart Lead (St Jude Medical Inc.) were the LV leads used for this study. The model 1056T or newer St Jude Medical LV leads were used when they became available. The use of bipolar leads was recommended. The RV lead was preferably placed at the RV apex, though other implantation sites were allowed. The LV lead was implanted transvenously via the coronary sinus, with a view to reach a lateral or postero-lateral LV segment. If these sites were unattainable, the lead was implanted elsewhere, as long as biventricular pacing was associated with a narrower QRS complex than RV pacing. An epicardial LV lead implantation was allowed if the transvenous approach was unsuccessful. Any commercially available ICD lead was allowed to be used, although the use of leads manufactured by St Jude Medical Inc. was encouraged.

# Atrioventricular synchronization

The individual AV delay was optimized in both study groups, for VDD and for DDD pacing, preferably using an electrocardiogram-based or echocardiogram-based method^1^. The dual and triple-chamber pulse generators as well as the corresponding ICD devices allowed the programming of different AV intervals for atrial-triggered ventricular stimulation (PV delay) and for AV sequential pacing (AV delay).

## Reference List

1. Butter C, Auricchio A, Stellbrink C et al. Effect of resynchronization therapy stimulation site on the systolic function of heart failure patients. Circulation 2001;104:3026–9.

# Randomization Algorithm

We did not apply a strict stratified randomization procedure. A dynamic randomization algorithm to balance treatment assignment in strata and their combinations was used. The randomization algorithm was based on a combination of several techniques and was constructed before patient randomization. For each new patient to be randomized, this dynamic randomization algorithm checked dynamically for imbalances in already performed allocations to the respective strata (and for combinations of these strata) and controlled for further imbalances taking into account only the strata to which the new patient belongs. Respectively, margins for noticeable allocation imbalance were pre-specified in the randomization software. Without observed noticeable imbalance, free 1:1 randomization was performed. Otherwise, biased coin randomization was implemented where the bias was increased with the amount of until so far detected imbalance in order to reduce the risk to further increase the imbalance. These techniques were implemented in a computer-program which took uniformly distributed random numbers in the unit interval [0; 1]. The confidential list of these random numbers was generated separately and documented before recruitment of the first patient.

# Additional Results

## Trial Profile

The trial profile (figure 1 of the main article) represents the CONSORT diagram of the BioPace study. In the following the flow of patients from level to level in the trial profile will be described. The respective levels are written in ***bold and italics***:

- The documentation starts at the ***enrollment*** level and comprises patients who have already given their informed consent for participation in the BioPace study. 1834 patients have been **scheduled for randomization**.

One patient had to be excluded in a Canadian center, because randomization did not work due to the time difference between Europe and Canada. Since the patient needed urgent care, the center implanted a device without randomization and declared end of study treatment.

1833 patients were **randomized, the first patient on May 27^th^, 2003, and the last patient on September 28^th^, 2007.** 915 patients were **allocated to biventricular pacing (BiV),** and 918 patients were **allocated to right ventricular pacing (RV)**.

13 patients in the BiV and 10 patients in the RV group had no legally valid written informed consent (WIC). These patients could neither be included in the safety analysis set nor in the ***intention-to-treat*** cohort (ITT).

- The ***ITT*** cohort therefore comprises 902 patients allocated to BiV (ITT-BiV) and 908 patients allocated to RV pacing (ITT-RV).

Considering the patients in the ITT-BiV group, no devices were implanted in 10 patients, one patient received a non-study BiV device, and 12 patients crossed over against randomization from BiV to RV, one of them receiving a non-study RV device.

Considering the patients in the ITT-RV group, no device was implanted in 8 patients, 16 patients received non-study RV devices, and 4 patients crossed over against randomization from RV to BiV. The patients who received non-study devices were excluded from our reporting on details concerning the implantation procedure (e.g. infection rate). For the time to event analyses however, these patients were kept at risk.

- In the vast majority of patients devices were implanted a few days after randomization. In 6 patients the initial implant attempt occurred later than 91 days after randomization, namely 99, 105, 124, 142, 155, and 161 days post randomization. The cohort with at least one attempt to implant a study device (***implantation of study device***) for BiV and RV pacing comprises 883 patients (Imp-BiV) and 891 patients (Imp-RV), respectively.

Considering the patients of the Imp-BiV group, device implantation was unsuccessful in 123 patients (13.9%):

- One patient (0.8%) died during the implant procedure.
- And in 122 patients (99.2%) the implantation of the LV lead failed in the first attempt.

Most common reasons for unsuccessful device implantation were inability to cannulate the coronary sinus in 35 patients (28.5%), inability to advance the lead into the coronary sinus in 25 patients (20.3%), high LV pacing threshold or phrenic nerve stimulation in 24 patients (19.5%), and no appropriate tributary target vein(s) in 15 patients (12.2%).

In the 4 patients who crossed over from RV to BiV study devices were successfully implanted.

14 of the 122 patients with failure to implant the LV lead in the first attempt received a BiV device in a second implant attempt, 10 of them within 3 months after the first implant attempt and 4 thereafter, namely 10 months, 3.3 years, 4.0 years, and 7.6 years after the first implant attempt. In the remaining 108 patients in whom no second attempt was undertaken or who had unsuccessful additional implant attempts RV devices were implanted. One of these patients received a RV non-study device. The 4 patients who had late and successful second LV lead implant attempts were temporarily provided with RV devices. Hence, 112 patients allocated in the BiV arm initially received RV devices.

- The ***hardware status after LV lead re-implant attempts within 3 months*** was BiV in 770 patients and RV in 1002 patients.

In one of the 4 patients who had crossed over from RV to BiV before the level ***implantation of study device*** the LV lead was deactivated right after implantation. In terms of device configuration this patient switched back from BiV to RV pacing despite being provided with a BiV device.

Based on a physician´s decision, that had been taken only 34 days after the initial RV device implant, another patient who belonged to the ITT-RV group and to the Imp-RV group crossed over from RV to BiV after the device had been upgraded by implanting a LV lead. According to the assessment of the Independent Event Adjudication Committee (IEAC), the latter upgrade from RV to BiV did not fulfill the protocol definition and was not clinically indicated.

- Thus, the numbers of patients remained unchanged from the level ***hardware status after LV lead re-implant attempts within 3 months*** to the level ***latest device configuration within 3 months after initial implantation***: BiV in 770 patients and RV in 1002 patients.

In order to facilitate the interpretation of the study result we describe the functionality of the devices, especially of the LV lead. We therefore assessed the ***functional status at 3 months after initial implantation.*** No problems of the pacing systems were found in the 1002 patients with RV device configuration. Of the 770 patients with BiV device configuration problems of the BiV devices occurred in 31 patients (4.0%):

- A pocket infection with perforation occurred in one patient (3.2%) 11 days after the initial implantation. The device was explanted after 1 month, end of study treatment was declared and the patient was lost to follow-up.
- A pacemaker-pocket MRSA-infection occurred in one patient (3.2%) and a RV study device was implanted at 18 days after initial implantation.
- And the LV lead was found to be dysfunctional in 29 patients (93.5%). Of those in one patient (3.2%) the LV lead was re-implanted within 2 days after initial implantation, thus ensuring LV capture. The functional status of the remaining 28 patients (90.3%) was considered RV, due to LV lead dislodgement in 7 patients (22.6%) and due to high LV threshold and/or phrenic nerve stimulation in 21 patients (67.7%).

Hence, including the patient who had a device pocket infection and who was downgraded to a RV study device, 29 patients switched at this level from BiV to RV.

Before reaching the level of 3 months after initial implantation, one patient who belonged to the ITT-BiV group and to the Imp-BiV group, died. The patient died during the implantation of the BiV device (as described at the level ***implantation of study device***). Another 29 patients died between implantation and 3 months of follow-up. Of those, 9 patients died while their latest device configuration was BiV. One of these patients died after the functional status of his device had changed from BiV to RV. And 20 patients died whose latest device configuration was RV.

Before reaching the level at 3 months after initial implantation, one patient who belonged to the ITT-BiV and the Imp-BiV group was lost to follow-up after his device had been explanted (as described in figure 1 at this level). In another 31 patients who were lost to follow up, the latest device configuration was BiV in 11 patients, one of whom had changed to the functional status RV. The remaining 20 losses to follow-up occurred in patients whose latest device configuration was RV.

- At the final level ***functional status at 3 months after initial implantation*** 722 patients were in the functional status BiV and 989 patients were in the functional status RV.

## Results in the *intention-to-treat* cohort

- All time to event outcomes were analyzed in the ***intention-to-treat*** cohort, the time at risk for each patient being started at the date of randomization (day zero). In April 2014 it became obvious that 635 events of the composite co-primary endpoint would have been observed by end May 2014. Hence, May 31^st^, 2014 was defined as the censoring date for time at risk for the composite co-primary endpoint.
- In order to give further pure descriptive insight in the results, subgroup analyses were added for the two co-primary endpoints regarding the pre-specified factors used for adjustment. In doing so LVEF & ICD stratification was concentrated to LVEF stratification because of the small sample size of the stratum LVEF ≤ 35%, and age was stratified in two strata using a cut-off of 75 years which was near the median age. LVEF was considered as a major prognostic factor which in addition was suggested to interact with the treatment group. Considering deaths that were not related to heart failure as competing events for which no advantage could be expected from BiV pacing, we assumed a modification of the effect size towards dilution with increasing LVEF. This gave the main distinction to the evolution of the study protocol and its amendments with major enlargement of patient recruitment and follow-up. The subgroup analyses were performed to explore, if the hazard ratios were consistent over subgroups or suggested an interaction with treatment. The presentation of the subgroup results in figures S1 and S2 included p-values for interaction. Note that these interaction p-values were descriptive and not adjusted for multiple testing. And since the interaction tests were not addressed in sample size planning, they were underpowered.
- Stratifying patients according to LVEF the hazard ratio to die or to be hospitalized due to heart failure (composite co-primary endpoint) of BiV vs. RV paced patients in the 152 patients with LVEF ≤ 35% (mean ventricular pacing percentage [VP%] at 1 month, 86.2%) was 0.83 (95% CI 0.54 to 1.26), for the 419 patients with LVEF between 36% and 50% (mean VP% at 1 month, 86.6%) the hazard ratio was 0.88 (95% CI 0.66 to 1.17) and for the 1239 patients with LVEF > 50% (mean VP% at 1 month, 89.2%) the hazard ratio was 0.90 (95% CI 0.74 to 1.08), (figure S1).
  Especially in the stratum of patients with LVEF ≤ 35% we had expected a HR clearly below 0.80. Also, in the stratum of patients with LVEF between 36% and 50% we had expected a HR around 0.80, while the HR of 0.90 in the stratum of patients with LVEF > 50% was within the expected range. Despite we were not able to demonstrate superiority of BiV compared to RV pacing there is also no evidence that there is definitely no positive effect in the subgroup of patients with LVEF > 50%. With respect to survival time the hazard ratio to die from any cause of BiV vs. RV paced patients in the 152 patients with LVEF ≤ 35% was 1.09 (95% CI 0.69 to 1.71), for the 419 patients with LVEF between 36% and 50% the hazard ratio was 0.92 (95% CI 0.67 to 1.26) and for the 1239 patients with LVEF > 50% the hazard ratio was 0.90 (95% CI 0.74 to 1.11), (figure S2).
- Analogically, we analyzed the key secondary endpoints time to cardiovascular death or hospitalization due to heart failure and time to cardiovascular death. Furthermore, we analyzed the post hoc defined composite endpoint time to heart failure manifestation resulting in death or hospitalization. These analyses were performed for explanatory purposes in the entire patient group and in the respective subgroups (figures S3, S4, S5, S6, S7, and S8). As expected, the HRs in the respective LVEF strata were somewhat lower in the composite consisting of cardiovascular death and heart failure hospitalization compared to the composite of death from any cause and hospitalization due to heart failure. Interestingly, the differences between the HRs of the respective LVEF strata are much the same in the two composite endpoints, the one containing death from any cause and the other containing cardiovascular death as a more specific component. Compared to these composite endpoints the heart failure manifestation endpoint appears to be even more specific since the HRs in all three LVEF strata are lower than in the other two composite endpoints. Of note, the differences between the HRs of the respective LVEF strata resemble strongly those in the other two composite endpoints. Taking all these findings together, they seem to suggest that there is a consistent positive effect of BiV pacing compared to RV pacing which, not surprisingly, is most obvious in the stratum with the lowest LVEF. On the other hand, this positive effect of BiV compared to RV pacing was obviously not strong enough to be proven in the co-primary endpoints we chose. From a retrospective point of view, it might appear questionable that we decided to investigate the effects of BiV compared to RV pacing in the co-primary endpoints we chose. Our main reason to choose these endpoints, and not the composite of time to heart failure manifestation resulting in death or hospitalization, was that we were too uncertain about the ability to correctly and thoroughly adjudicate deaths due to heart failure. We suspected that too many of these events would not be recognized and classified as such.
- Looking at interaction we found a possible interaction between sex and treatment in the composite endpoint time to cardiovascular death or heart failure hospitalization and in the endpoint time to heart failure manifestation resulting in death or hospitalization (figures S4 and S8). These results suggest that the difference between RV and BiV pacing could be smaller in women than in men. However, there was no evidence of heterogeneity of treatment effect by subgroup for any of the subgroups tested.
- As shown in figure S9 and table S1 mean ventricular pacing percentage was between 85% and 95% at most of the study visits. In addition, it can also be seen that the mean ventricular pacing percentage of RV paced patients was by some 6% lower than in the BiV arm. The differences in ventricular pacing percentage between both study arms differed only slightly between the LVEF strata (figures S9, S10, S11, and S12 and tables S1, S2, S3, and S4).

## Results in the cohort with implantation of study devices

- In total, LV lead implantation was successful in the first attempt in 760 out of 883 patients in whom it was attempted. Including second implant attempts that were performed within the first three months after the first attempt, a total of 770 out of 883 patients had received a biventricular device. The overall success rate after the first and second attempt was hence 86.1% and 87.2%, respectively. When analyzing the data, the time line of three months was arbitrarily defined on the one hand to include most of the second implant attempts and on the other hand to describe the functional status of the pacing systems at a rather early stage of the study. If including also later second re-attempts to implant the LV lead the formal overall success rate was 87.7%.
- Right ventricular pacing devices could be implanted in the first attempt in all 891 patients who were scheduled for it (success rate = 100%).
- Of the 123 patients with unsuccessful first implant attempts 8, 33 and 82 were in the subgroups with a LVEF ≤ 35%, 35% < LVEF ≤ 50%, and LVEF > 50%, respectively.
- Fluoroscopy times during the first implant procedure were reported in 846 patients (95.8%) where implantation of a BiV device and in 827 patients (92.8%) where implantation of a RV device was attempted, irrespective of whether the first attempt was successful or not. The median fluoroscopy times were 19.0 minutes and 4.0 minutes, respectively.

## Results on functional status at three months after initial implantation

- We used the LV pacing threshold values to verify efficient LV lead function at three months after initial implantation. In 7 of the 722 patients with biventricular configuration only values at pre-hospital-discharge were collected. In these 7 patients the LV leads were functioning at pre-discharge. Since we did not consider the pre-hospital-discharge LV lead thresholds as valid to assess long-lasting LV lead function we analyzed LV pacing thresholds at one month post implantation or thereafter. These values were available in the remaining 715 patients and are displayed in table S5. Based on a post-hoc definition we considered LV leads with pacing thresholds characterized as a voltage amplitude of > 3.0 V **in combination** with a pulse duration of > 1.0 ms (***bold and italics***) as potentially not reliably functioning. This was the case in 9 of 722 patients (1.25%) who were on biventricular pacing at three months after the initial implantation. However, since functioning is documented, albeit at a high pacing threshold, these patients were not considered as crossovers to RV pacing.
- 55 patients, all with functioning LV leads at 3 months after initial implantation, switched from BiV to RV pacing during the subsequent study period (see table S6). Of note, 28 of these 55 switches (51%) occurred during the first 12 months after initial implantation. The table also shows the number and time points of switches from RV to BiV. It is noteworthy that 4 of these 52 switches represent late successful second re-implant attempts of the LV lead in patients who had initially been randomized to BiV pacing. These 4 patients were already reported earlier (see section .1 of this chapter). One of these 4 patients switched from RV to BiV at 10 months after first implant, the other 3 during their later follow-up at 40, 48, and 91 months. In addition, 2 of the 52 switches represent a) one repositioning of a dislocated LV lead after 18 months and b) one programming to BiV pacing after 36 months with borderline functioning of a partially dislocated LV lead. Hence, a total of 46 patients who had initially been randomized to RV pacing, switched to BiV pacing, 6 (13%) of them during the first year of follow-up and 40 (87%) thereafter. The independent event adjudication committee (IEAC) adjudicated 3 of these 46 upgrades as in accordance with the study protocol, 14 were assessed as not conform to the study protocol but clinically indicated and 29 were assessed neither as conform to the study protocol nor as clinically indicated. Interestingly, one of the 29 patients (patient with ** in the flow chart) was initially randomized to RV pacing. Against randomization he received a BiV device in the first implant attempt, his LV lead was however inactivated right after implant and it was reactivated at 19 months of follow-up so that he was under BiV pacing for the rest of the study period.
- From the perspective of the intention-to-treat cohort a total of 208 patients crossed over from BiV to RV pacing. As described in the study flow chart, there were 12 crossovers before the first implant attempt, 112 patients crossed over due to failure of the first implant attempt and another 29 patients switched from BiV to RV pacing due to malfunction of the LV lead which occurred during the first three months after implantation. Thereafter, another 55 patients crossed over from BiV to RV pacing. Likewise, a total of 50 patients of the intention-to-treat cohort crossed over from RV to BiV pacing. Four of these patients crossed over before the first implant attempt. One of them switched back immediately after implantation, due to inactivation of the LV lead. One patient was upgraded to BiV pacing within three months after initial implantation. Thereafter, another 46 patients switched from RV to BiV pacing (see trial profile and table S6).

## Sensitivity Analyses

Of the 1810 intention-to-treat cohort patients, 12 patients of the BiV group and 8 patients of the RV group did not receive a device (19 not implanted, one explanted right after implantation and not re-implanted). Excluding these patients, a sensitivity analysis of the primary endpoints in the remaining 1790 patients (890 in the BiV group and 900 in the RV group), resulted in 345 versus 363 composite events (HR, 0.875; 95% CI, 0.753 to 1.016; p=0.0805) and 304 versus 307 deaths (HR, 0.923; 95% CI, 0.786 to 1.084; p=0.3265), respectively.

During the follow-up until 3 months after the initial implant attempt, there were 153 crossovers from BiV to RV pacing and 4 crossovers from RV to BiV pacing (figure 1 with attention on legend **). Taking this into account and excluding patients without functional devices at 3 months after initial implantation, a sensitivity analysis was performed. The analysis was performed grouped by the functional status at 3 months after initial implantation (similar to an as-treated analysis) of the primary endpoints in the remaining 1728 patients, 723 in the BiV group (1 of them with a non-study device) and 1005 in the RV group (16 of them with a non-study device). This resulted in 283 versus 395 composite events (HR, 0.943; 95% CI, 0.807 to 1.102; p=0.4625) and 247 versus 334 deaths (HR, 1.001; 95% CI, 0.846 to 1.184; p=0.9902), respectively. It has to be taken into account that a selection bias may have influenced the results in favor of RV pacing. In a portion of patients who switched from BiV to RV (n=153, n=146 of them included in this sensitivity analysis) or from RV to BiV (n=4, all of them included in this sensitivity analysis) it cannot be excluded that the decision to switch was influenced by the characteristics of those patients which usually go along with a favorable or unfavorable prognosis, respectively. E.g., the average QRS duration in those patients who switched from BiV to RV pacing was lower than the average QRS duration of all patients randomized to BiV pacing on the one hand. On the other hand, those patients who switched from RV to BiV pacing had in average a longer QRS duration than the entire cohort of patients that had been randomized to RV pacing. This selection bias could not be excluded, since the implanting physicians were of course not blinded for treatment allocation and overall clinical data of the patients.

## Safety issues

Detailed information about serious and non-serious adverse events classified according to whether procedure, system, device, lead or infection related is displayed in tables S7-S10.

# Figures and Tables


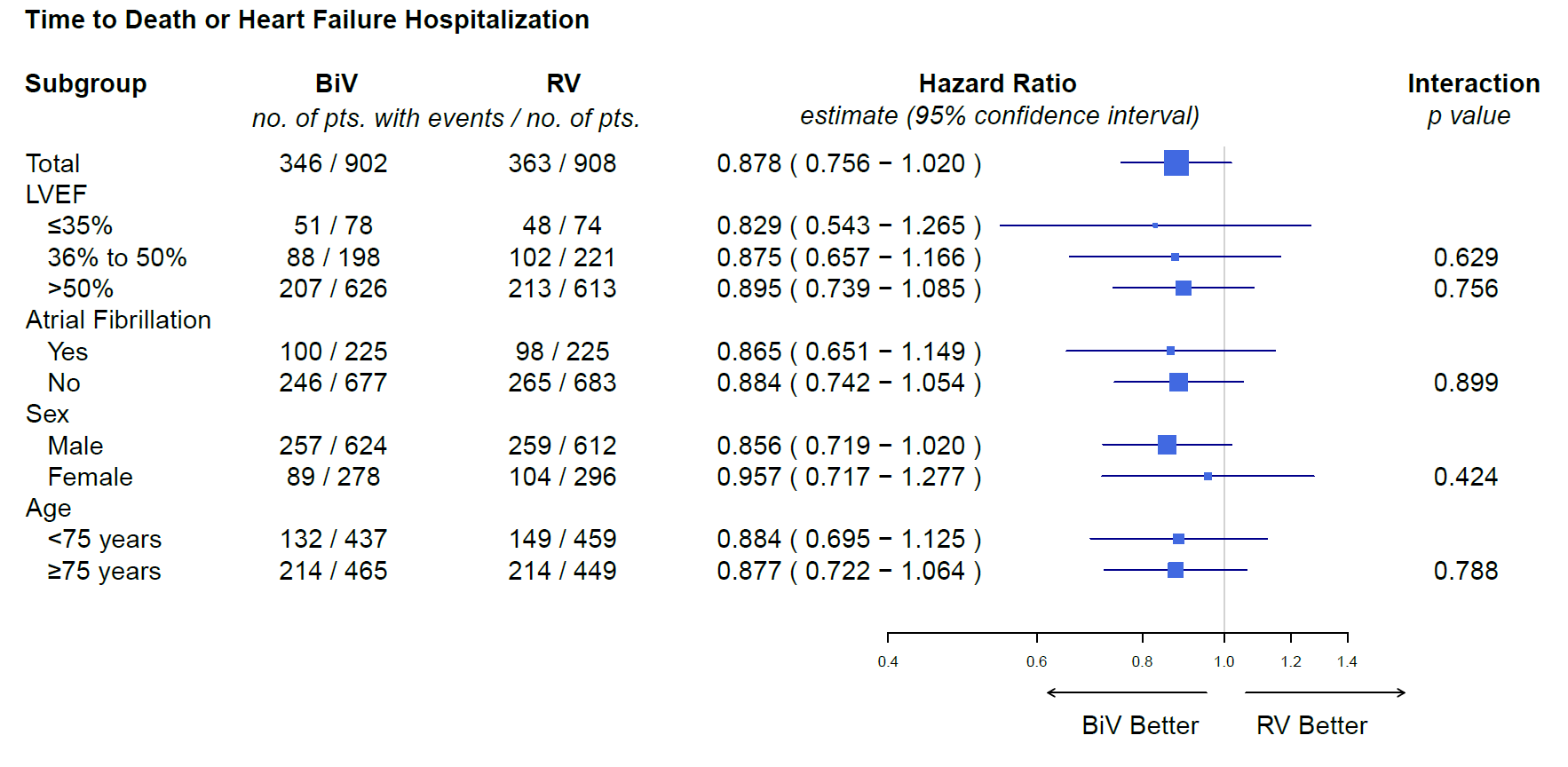


**Figure S1: Time to death or hospitalization due to heart failure**

Subgroup analyses for strata according to the prognostic factors pre-defined for adjustment in the analysis are shown in a Forest plot. Exploratory p-values for interaction with treatment group are included. The confidence intervals reveal considerable consistency and no interaction is visible.


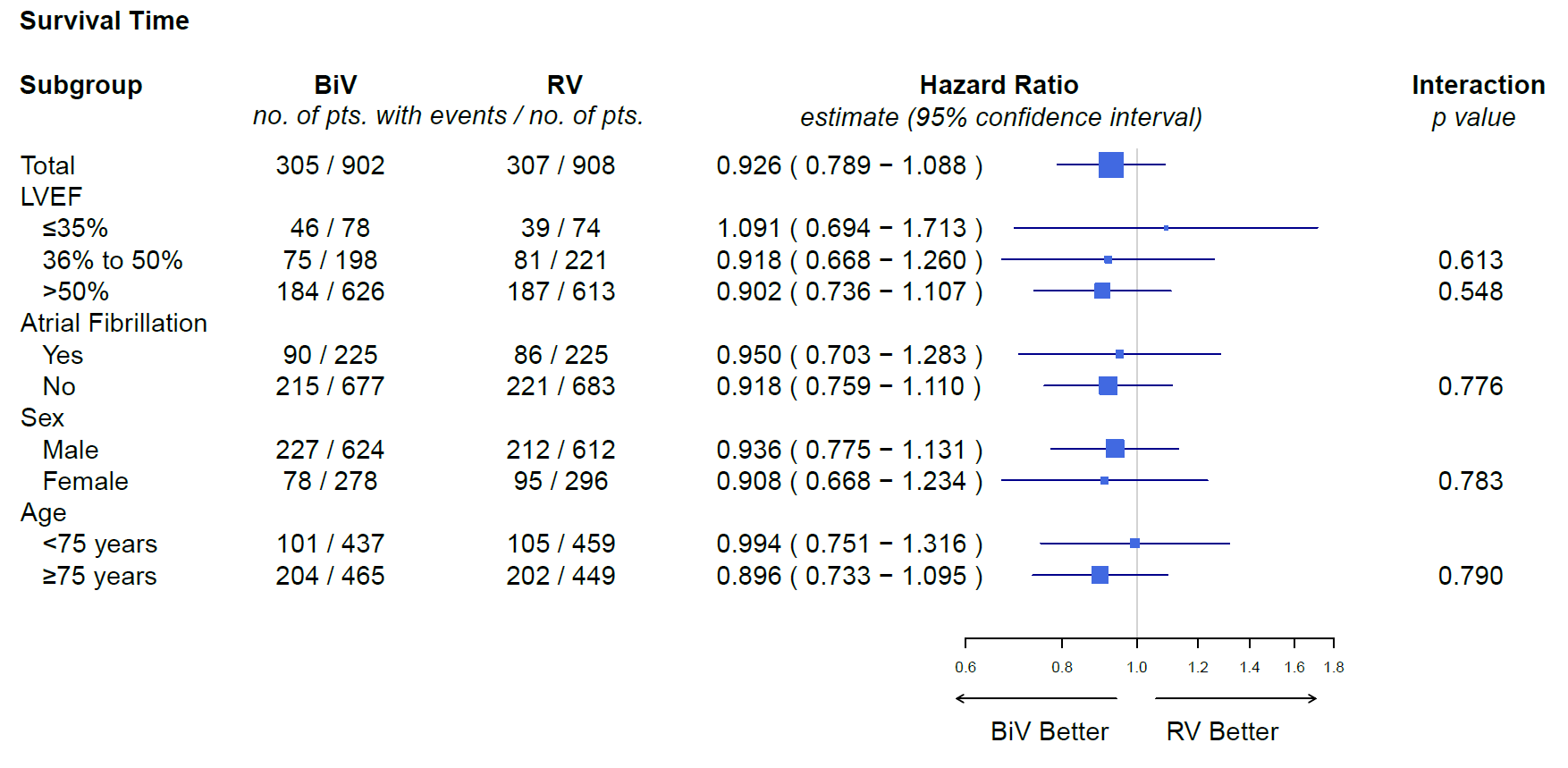
**Figure S2: Survival Time**

Subgroup analyses for strata according to the prognostic factors pre-defined for adjustment in the analysis are shown in a Forest plot. Exploratory p-values for interaction with treatment group are included. The confidence intervals reveal considerable consistency and no interaction is visible.


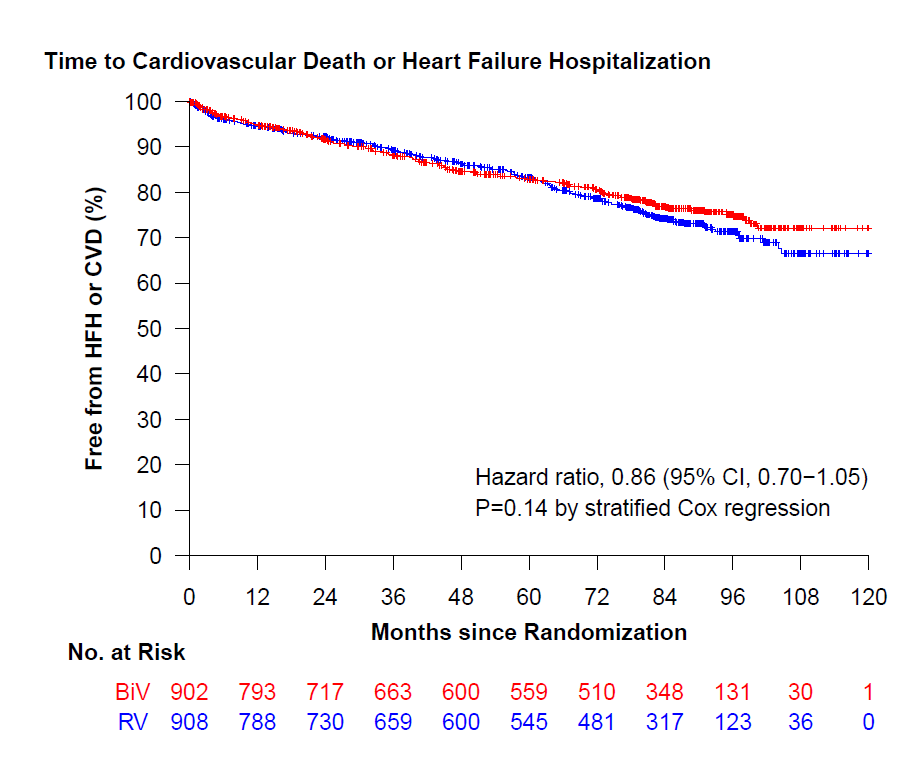


**Figure S3: Time to cardiovascular death or hospitalization due to heart failure**

Kaplan-Meier estimates with number of patients at risk of the treatment are shown over 10 years of follow-up for comparison of biventricular pacing (BiV) with right ventricular pacing (RV).


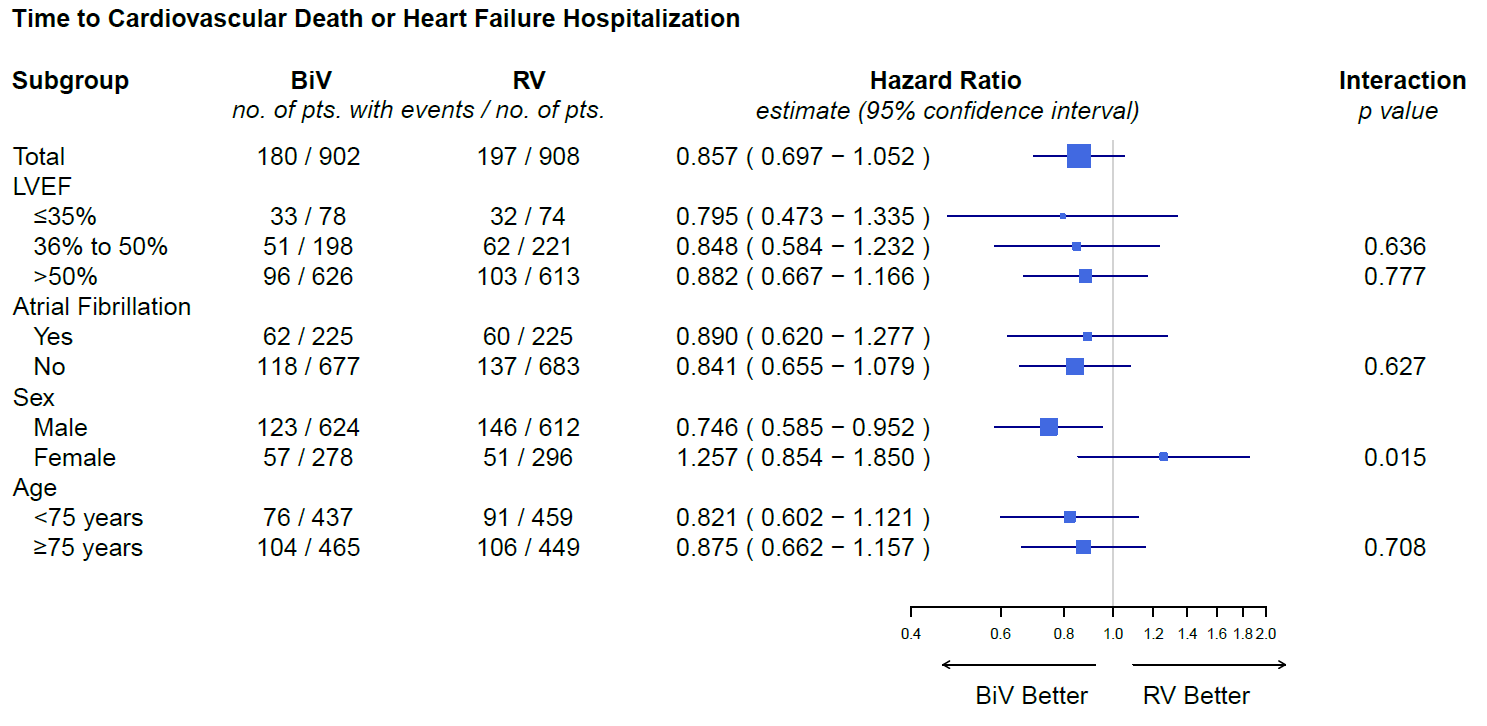
**Figure S4:** **Time to cardiovascular death or hospitalization due to heart failure**

Subgroup analyses for strata according to the prognostic factors pre-defined for adjustment in the analysis are shown in a Forest plot. Exploratory p-values for interaction with treatment group are included. The confidence intervals reveal considerable consistency while it is suggested that the result of BiV pacing could be worse in females than in males.


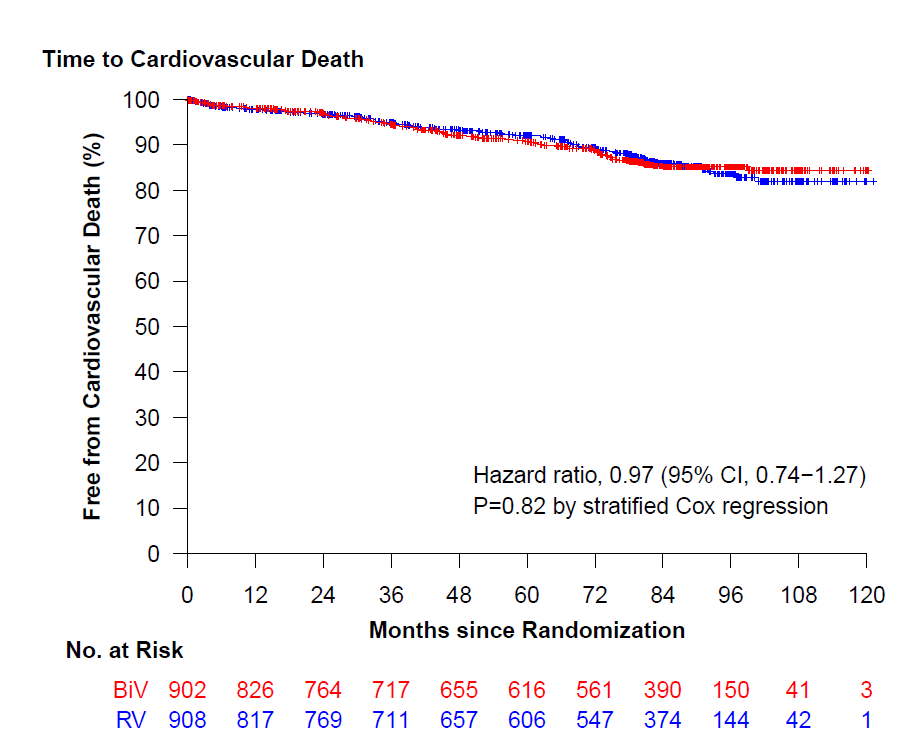


**Figure S5:** **Time to cardiovascular death**

Kaplan-Meier estimates with number of patients at risk of the treatment are shown over 10 years of follow-up for comparison of biventricular pacing (BiV) with right ventricular pacing (RV).


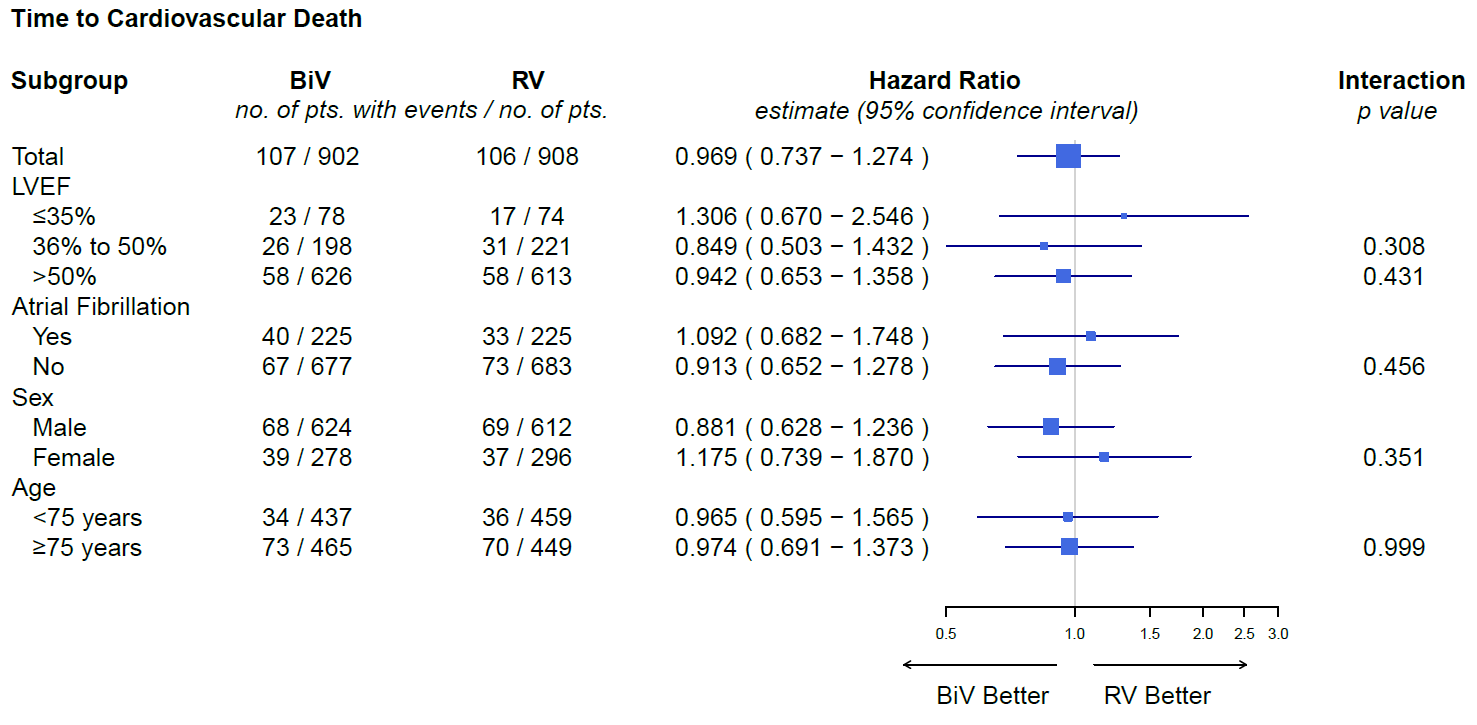


**Figure S6:** **Time to cardiovascular death**

Subgroup analyses for strata according to the prognostic factors pre-defined for adjustment in the analysis are shown in a Forest plot. Exploratory p-values for interaction with treatment group are included. The confidence intervals reveal considerable consistency while it is suggested that the result of BiV pacing could interestingly be worse in patients with LVEF≤35% and in females.


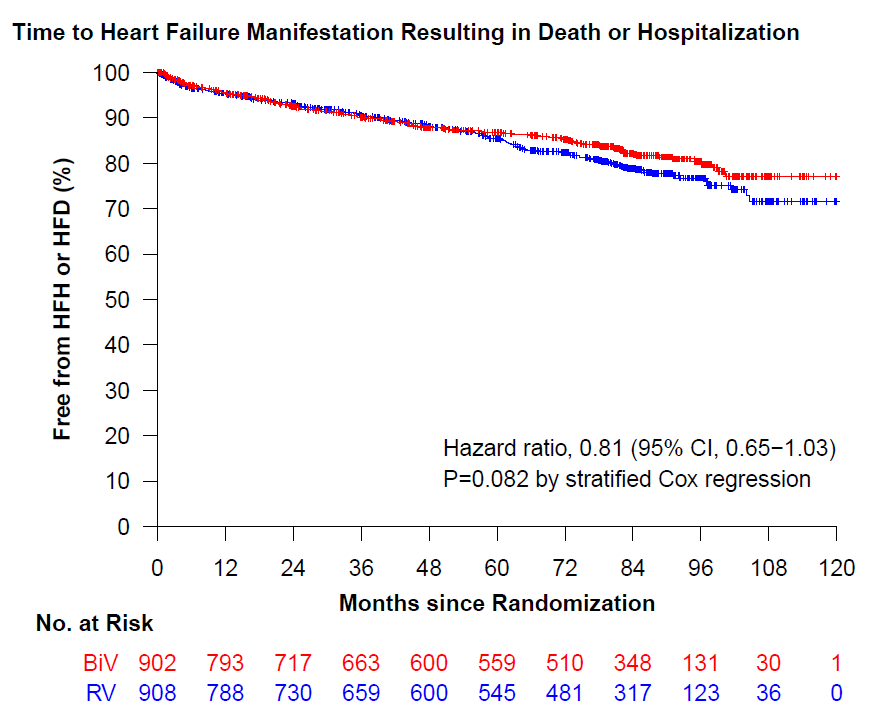


**Figure S7:** **Time to heart failure manifestation resulting in death or hospitalization**

Kaplan-Meier estimates with number of patients at risk of the treatment are shown over 10 years of follow-up for comparison of biventricular pacing (BiV) with right ventricular pacing (RV).


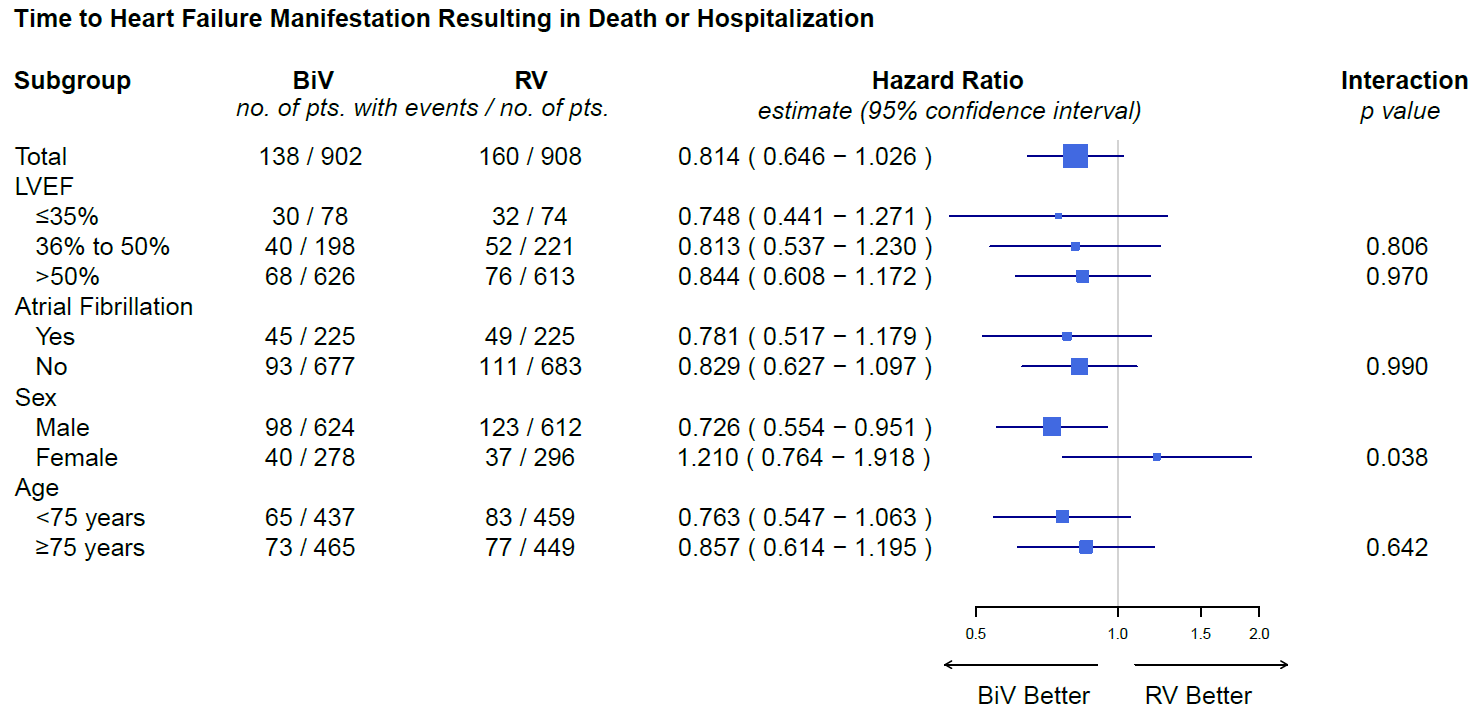


**Figure S8:** **Time to heart failure manifestation resulting in death or hospitalization**

Subgroup analyses for strata according to the prognostic factors pre-defined for adjustment in the analysis are shown in a Forest plot. Exploratory p-values for interaction with treatment group are included. The confidence intervals reveal considerable consistency while it is suggested that the result of BiV pacing could be worse in females than in males.


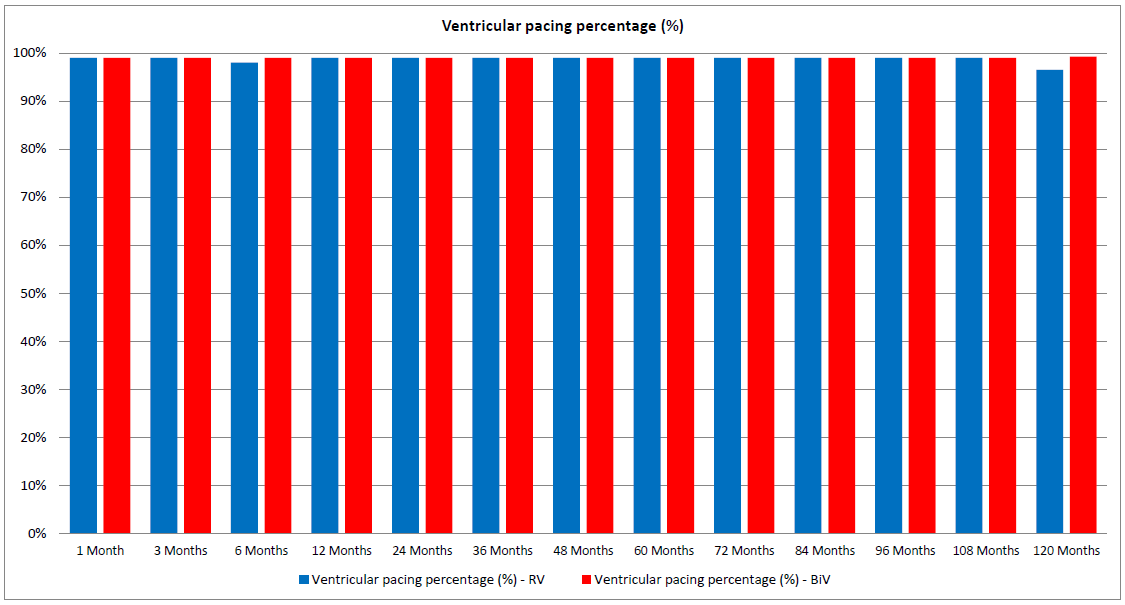


**Figure S9: Median ventricular pacing percentages in both study arms during the study**

**Table S1: Median ventricular pacing percentages in both study arms during the study**

| **Follow-up time — months** | **1** | **3** | **6** | **12** | **24** | **36** | **48** | **60** | **72** | **84** | **96** | **108** | **120** |
| --- | --- | --- | --- | --- | --- | --- | --- | --- | --- | --- | --- | --- | --- |
| **Ventricular pacing percentage — %: RV** | 99.00 | 99.00 | 98.00 | 99.00 | 99.00 | 99.00 | 99.00 | 99.00 | 99.00 | 99.00 | 99.00 | 99.00 | 96.50 |
| **Ventricular pacing percentage — %: BiV** | 99.00 | 99.00 | 99.00 | 99.00 | 99.00 | 99.00 | 99.00 | 99.00 | 99.00 | 99.00 | 99.00 | 99.00 | 99.25 |
| **Difference in VP percentages — %: BiV − RV** | 0 | 0 | 1 | 0 | 0 | 0 | 0 | 0 | 0 | 0 | 0 | 0 | 2.75 |
| **Number of patients: RV** | 803 | 167 | 779 | 760 | 715 | 645 | 570 | 515 | 451 | 286 | 102 | 36 | 2 |
| **Number of patients: BiV** | 818 | 192 | 783 | 759 | 711 | 630 | 557 | 496 | 462 | 291 | 114 | 32 | 2 |
| **Number of patients: cumulative** | 1,621 | 359 | 1,562 | 1,519 | 1,426 | 1,275 | 1,127 | 1,011 | 913 | 577 | 216 | 68 | 4 |

Presentation of median ventricular pacing percentages and of the number of patients in whom ventricular pacing percentages were available at the respective visits.


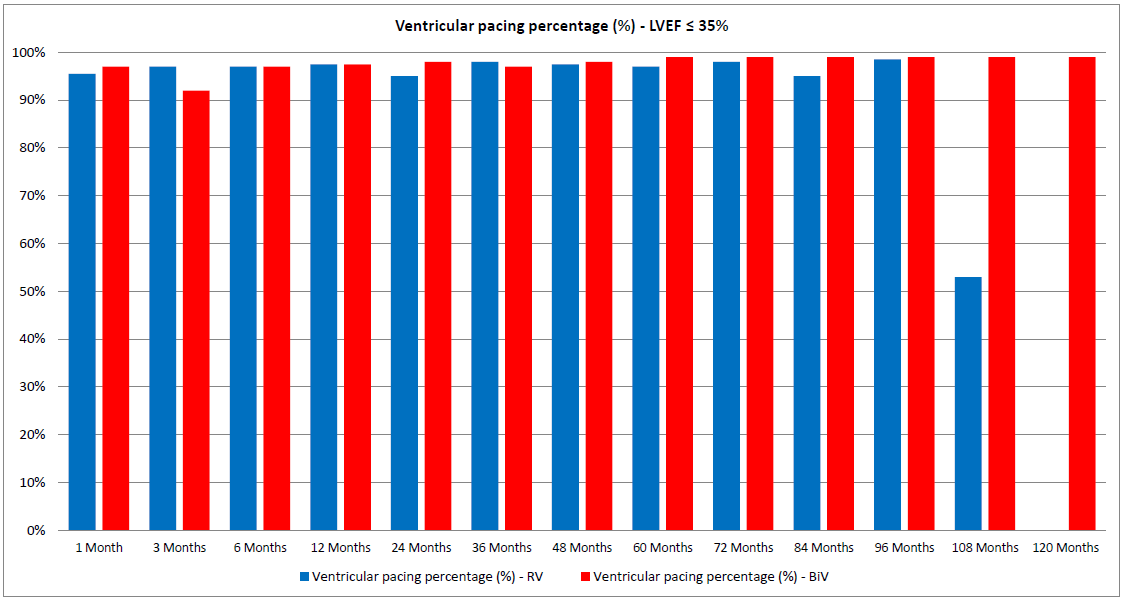


**Figure S10: Median ventricular pacing percentages in both study arms during the study for LVEF ≤ 35%**

**Table S2: Median ventricular pacing percentages in both study arms during the study for LVEF ≤ 35%**

| **Follow-up time — months** | **1** | **3** | **6** | **12** | **24** | **36** | **48** | **60** | **72** | **84** | **96** | **108** | **120** |
| --- | --- | --- | --- | --- | --- | --- | --- | --- | --- | --- | --- | --- | --- |
| **Ventricular pacing percentage — %: RV** | 95.50 | 97.00 | 97.00 | 97.50 | 95.00 | 98.00 | 97.50 | 97.00 | 98.00 | 95.00 | 98.50 | 53.00 |  |
| **Ventricular pacing percentage — %: BiV** | 97.00 | 92.00 | 97.00 | 97.50 | 98.00 | 97.00 | 98.00 | 99.00 | 99.00 | 99.00 | 99.00 | 99.00 | 99.00 |
| **Difference in VP percentages — %: BiV − RV** | 1.50 | -5.00 | 0.00 | 0.00 | 3.00 | -1.00 | 0.50 | 2.00 | 1.00 | 4.00 | 0.50 | 46.00 |  |
| **Number of patients: RV** | 70 | 20 | 61 | 58 | 49 | 35 | 28 | 23 | 19 | 11 | 4 | 1 | 0 |
| **Number of patients: BiV** | 69 | 18 | 56 | 60 | 53 | 39 | 36 | 26 | 20 | 11 | 5 | 1 | 1 |
| **Number of patients: cumulative** | 139 | 38 | 117 | 118 | 102 | 74 | 64 | 49 | 39 | 22 | 9 | 2 | 1 |

Presentation of median ventricular pacing percentages and of the number of patients in whom ventricular pacing percentages were available at the respective visits.


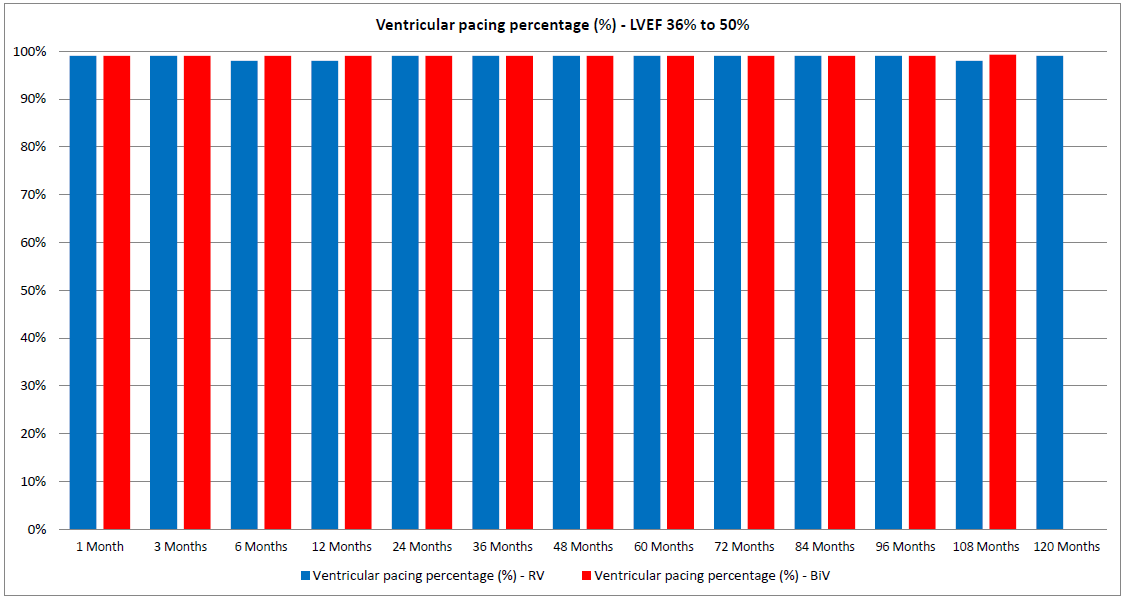


**Figure S11: Median ventricular pacing percentages in both study arms during the study for LVEF 36% to 50%**

**Table S3: Median ventricular pacing percentages in both study arms during the study for LVEF 36% to 50%**

| **Follow-up time — months** | **1** | **3** | **6** | **12** | **24** | **36** | **48** | **60** | **72** | **84** | **96** | **108** | **120** |
| --- | --- | --- | --- | --- | --- | --- | --- | --- | --- | --- | --- | --- | --- |
| **Ventricular pacing percentage — %: RV** | 99.00 | 99.00 | 98.00 | 98.00 | 99.00 | 99.00 | 99.00 | 99.00 | 99.00 | 99.00 | 99.00 | 98.00 | 99.00 |
| **Ventricular pacing percentage — %: BiV** | 99.00 | 99.00 | 99.00 | 99.00 | 99.00 | 99.00 | 99.00 | 99.00 | 99.00 | 99.00 | 99.00 | 99.30 |  |
| **Difference in VP percentages — %: BiV − RV** | 0.00 | 0.00 | 1.00 | 1.00 | 0.00 | 0.00 | 0.00 | 0.00 | 0.00 | 0.00 | 0.00 | 1.30 |  |
| **Number of patients: RV** | 197 | 42 | 187 | 175 | 167 | 149 | 121 | 109 | 95 | 63 | 21 | 9 | 1 |
| **Number of patients: BiV** | 171 | 47 | 171 | 157 | 153 | 133 | 109 | 101 | 96 | 60 | 23 | 11 | 0 |
| **Number of patients: cumulative** | 368 | 89 | 358 | 332 | 320 | 282 | 230 | 210 | 191 | 123 | 44 | 20 | 1 |

Presentation of median of ventricular pacing percentages and of the number of patients in whom ventricular pacing percentages were available at the respective visits.


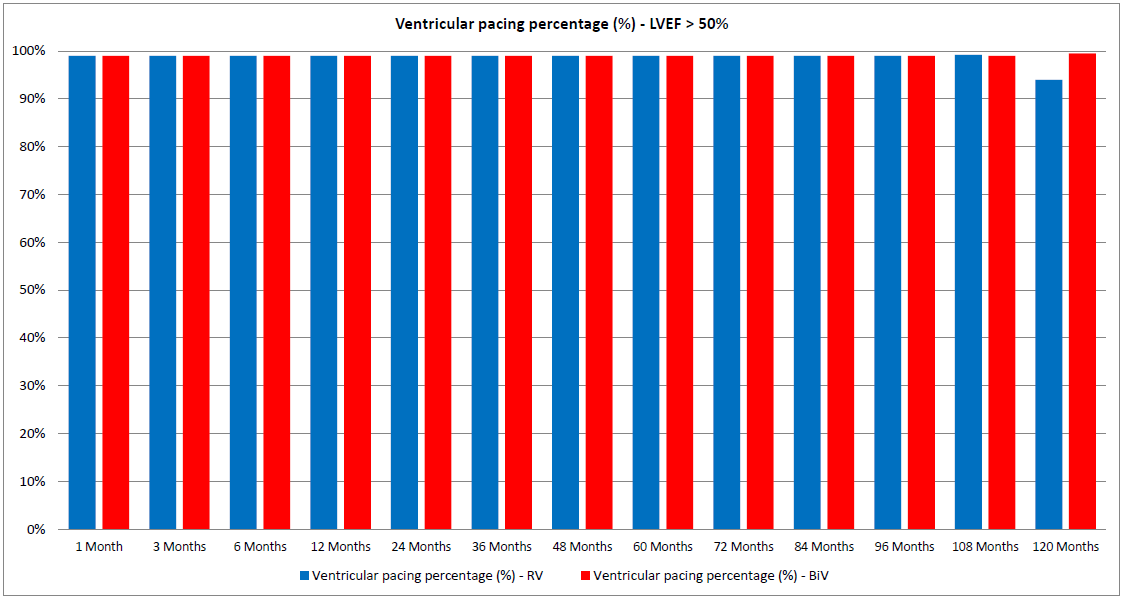


**Figure S12: Median ventricular pacing percentages in both study arms during the study for LVEF > 50%**

**Table S4: Median ventricular pacing percentages in both study arms during the study for LVEF > 50%**

| **Follow-up time — months** | **1** | **3** | **6** | **12** | **24** | **36** | **48** | **60** | **72** | **84** | **96** | **108** | **120** |
| --- | --- | --- | --- | --- | --- | --- | --- | --- | --- | --- | --- | --- | --- |
| **Ventricular pacing percentage — %: RV** | 99.00 | 99.00 | 99.00 | 99.00 | 99.00 | 99.00 | 99.00 | 99.00 | 99.00 | 99.00 | 99.00 | 99.25 | 94.00 |
| **Ventricular pacing percentage — %: BiV** | 99.00 | 99.00 | 99.00 | 99.00 | 99.00 | 99.00 | 99.00 | 99.00 | 99.00 | 99.00 | 99.00 | 99.00 | 99.50 |
| **Difference in VP percentages — %: BiV − RV** | 0.00 | 0.00 | 0.00 | 0.00 | 0.00 | 0.00 | 0.00 | 0.00 | 0.00 | 0.00 | 0.00 | -0.25 | 5.50 |
| **Number of patients: RV** | 536 | 105 | 531 | 527 | 499 | 461 | 421 | 383 | 337 | 212 | 77 | 26 | 1 |
| **Number of patients: BiV** | 578 | 127 | 556 | 542 | 505 | 458 | 412 | 369 | 346 | 220 | 86 | 20 | 1 |
| **Number of patients: cumulative** | 1,114 | 232 | 1,087 | 1,069 | 1,004 | 919 | 833 | 752 | 683 | 432 | 163 | 46 | 2 |

Presentation of median of ventricular pacing percentages and of the number of patients in whom ventricular pacing percentage were available at the respective visits.

**Table S5: Functioning of LV lead at three months after initial implantation**

| **Pulse duration** | **≤ 0.5 ms** | **0.6-1.0 ms** | **1.1 – 1.2 ms** | **>1.2 ms** |
| --- | --- | --- | --- | --- |
| **Amplitude ≤ 1.0 V** | 285 (39.9) | 45 (6.3) | 4 (0.6) | 36 (5.0) |
| **Amplitude 1.1 – 3 V** | 172 (24.1) | 77 (10.8) | 7 (1.0) | 49 (6.9) |
| **Amplitude 3.1 – 6 V** | 12 (1.7) | 16 (2.2) | ***2 (0.3)*** | ***7 (1.0)*** |
| **Amplitude > 6 V** | 1 (0.1) | 2 (0.3) | 0 | 0 |

Number of patients (%)

For long-lasting LV lead function in the biventricular group functioning at three months after initial implantation LV pacing thresholds at one month post implantation or thereafter were analyzed.

In 715 of the 722 patients with biventricular configuration at three months after initial implantation the amplitude and pulse duration were available. Pacing thresholds > 3.0 V in combination with pulse durations > 1.0 ms were considered as borderline so that these 9 leads were considered as potentially not reliably functioning. Long-lasting LV lead function was expected in 706 patients.

In the remaining 7 patients only the information that the LV leads were functioning at pre-hospital-discharge was available.

**Table S6: Number of patients with crossover from BiV to RV pacing and number of patients with crossover from RV to BiV pacing by time of crossover later than three months after initial implantation**

| **Time of crossover** | **Number of patients switched from BiV to RV** | **Number of patients switched from RV to BiV** |
| --- | --- | --- |
| month 4 – 6 | 17 | 4 |
| month 7 – 12 | 11 | 3 |
| month 13 – 24 | 3 | 7 |
| month 25 – 36 | 10 | 7 |
| month 37 – 48 | 5 | 10 |
| month 49 – 60 | 3 | 3 |
| month 61 – 72 | 3 | 5 |
| month 73 – 84 | 2 | 9 |
| month 85 – 96 | 0 | 2 |
| month 97 – 108 | 1 | 1 |
| month 109 – 120 | 0 | 1 |
| **Total** | **55** | **52** |

**Table S7: Serious adverse events – number of events**

|  | **Randomized Group** | | | | | |
| --- | --- | --- | --- | --- | --- | --- |
|  | **Biventricular** | | **Right ventricular** | | **Total** | |
|  | **N=2,111** | | **N=1,939** | | **N=4,050** | |
|  | **N** | **%** | **N** | **%** | **N** | **%** |
| **Procedure related^[[1]](#footnote-1)^** | 115 | 5.45 | 72 | 3.71 | 187 | 4.62 |
| **System related^[[2]](#footnote-2)^** | 9 | 0.43 | 7 | 0.36 | 16 | 0.40 |
| **Device related^[[3]](#footnote-3)^** | 28 | 1.33 | 12 | 0.62 | 40 | 0.99 |
| **Total lead-related^[[4]](#footnote-4)^** | 87 | 4.12 | 54 | 2.78 | 141 | 3.48 |
| - **RA lead related** | 11 | 0.52 | 14 | 0.72 | 25 | 0.62 |
| - **RV lead related** | 30 | 1.42 | 30 | 1.55 | 60 | 1.48 |
| - **LV lead related** | 43 | 2.04 | 1 | 0.05 | 44 | 1.09 |
| - **Lead related unspecified** | 3 | 0.14 | 9 | 0.46 | 12 | 0.30 |
| **Infections related to the device and/or lead** | 19 | 0.90 | 16 | 0.83 | 35 | 0.86 |
| **Other^[[5]](#footnote-5)^** | 1,853 | 87.78 | 1,778 | 91.70 | 3,631 | 89.65 |

**Table S8: Non serious adverse events – number of events**

|  | **Randomized Group** | | | | | |
| --- | --- | --- | --- | --- | --- | --- |
|  | **Biventricular** | | **Right ventricular** | | **Total** | |
|  | **N=856** | | **N=728** | | **N=1,584** | |
|  | **N** | **%** | **N** | **%** | **N** | **%** |
| **Procedure related^[[6]](#footnote-6)^** | 83 | 9.70 | 37 | 5.08 | 120 | 7.58 |
| **System related^[[7]](#footnote-7)^** | 3 | 0.35 | 2 | 0.27 | 5 | 0.32 |
| **Device related7** | 22 | 2.57 | 26 | 3.57 | 48 | 3.03 |
| **Total lead-related^[[8]](#footnote-8)^** | 113 | 13.20 | 27 | 3.71 | 140 | 8.84 |
| - **RA lead related** | 10 | 1.17 | 8 | 1.10 | 18 | 1.14 |
| - **RV lead related** | 12 | 1.40 | 8 | 1.10 | 20 | 1.26 |
| - **LV lead related** | 86 | 10.05 | 8 | 1.10 | 94 | 5.93 |
| - **Lead related unspecified** | 5 | 0.58 | 3 | 0.41 | 8 | 0.51 |
| **Infections related to the device and/or lead** | 3 | 0.35 | 2 | 0.27 | 5 | 0.32 |
| **Other^[[9]](#footnote-9)^** | 632 | 73.83 | 634 | 87.09 | 1,266 | 79.92 |

**Table S9: Serious adverse events – number of patients**

|  | **Randomized Group** | | | | | |
| --- | --- | --- | --- | --- | --- | --- |
|  | **Biventricular** | | **Right ventricular** | | **Total** | |
|  | **N=902** | | **N=908** | | **N=1,810** | |
|  | **N** | **%** | **N** | **%** | **N** | **%** |
| **Procedure related** | 93 | 10.31 | 69 | 7.60 | 162 | 8.95 |
| **System related** | 9 | 1.00 | 5 | 0.55 | 14 | 0.77 |
| **Device related** | 22 | 2.44 | 10 | 1.10 | 32 | 1.77 |
| **Total lead-related** | 81 | 8.98 | 46 | 5.07 | 127 | 7.02 |
| - **RA lead related** | 11 | 1.22 | 12 | 1.32 | 23 | 1.27 |
| - **RV lead related** | 29 | 3.22 | 24 | 2.64 | 53 | 2.93 |
| - **LV lead related** | 38 | 4.21 | 1 | 0.11 | 39 | 2.15 |
| - **Lead related unspecified** | 3 | 0.33 | 9 | 0.99 | 12 | 0.66 |
| **Infections related to the device and/or lead** | 17 | 1.88 | 13 | 1.43 | 30 | 1.66 |
| **Other** | 624 | 69.18 | 603 | 66.41 | 1,227 | 67.79 |

**Table S10: Non serious adverse events – number of patients**

|  | **Randomized Group** | | | | | |
| --- | --- | --- | --- | --- | --- | --- |
|  | **Biventricular** | | **Right ventricular** | | **Total** | |
|  | **N=902** | | **N=908** | | **N=1,810** | |
|  | **N** | **%** | **N** | **%** | **N** | **%** |
| **Procedure related** | 75 | 8.31 | 35 | 3.85 | 110 | 6.08 |
| **System related** | 3 | 0.33 | 2 | 0.22 | 5 | 0.28 |
| **Device related** | 18 | 2.00 | 21 | 2.31 | 39 | 2.15 |
| **Total lead-related** | 99 | 10.98 | 26 | 2.86 | 125 | 6.91 |
| - **RA lead related** | 9 | 1.00 | 8 | 0.88 | 17 | 0.94 |
| - **RV lead related** | 12 | 1.33 | 8 | 0.88 | 20 | 1.10 |
| - **LV lead related** | 73 | 8.09 | 8 | 0.88 | 81 | 4.48 |
| - **Lead related unspecified** | 5 | 0.55 | 2 | 0.22 | 7 | 0.39 |
| **Infections related to the device and/or lead** | 2 | 0.22 | 2 | 0.22 | 4 | 0.22 |
| **Other** | 324 | 35.92 | 312 | 34.36 | 636 | 35.14 |

1. All procedure related serious events occurred up to 30 days after implant and include: acute pulmonary edema, atrial fibrillation, cardiac arrest, cardiac perforation, chest pain, coronary sinus dissection, diaphragmatic nerve stimulation, dyspnea, elevated pacing threshold, exit block, fever, lead dislodgment, lead replacement, lead repositioning, lead dysfunction, loss of capture, loss of sensing, pain, hematoma, bleeding, device dysfunction, device pocket decubitus, device pocket revision, device pocket swelling, pulmonary embolism, sickness, subclavian vein thrombosis, syncope, tachycardia. [↑](#footnote-ref-1)
2. System related serious events include: elevated pacing threshold, exteriorization of the pacemaker, myocardial infarction, upgrade to CRT-D/CRT-P, lead dislodgment, worsening of HF, device pocket revision, lead dysfunction. [↑](#footnote-ref-2)
3. Device related serious events include: pain, device pocket edema, device pocket erosion, device explantation, device dysfunction, device pocket hematoma, device pocket perforation, device pocket decubitus, and device pocket revision. [↑](#footnote-ref-3)
4. Lead related serious events include: lead fracture, lead dislodgement, lead revision, loss of capture, lead dysfunction, loss of sensing, elevated pacing threshold, oversensing, undersensing, lead externalization, pacemaker syndrome, and diaphragmatic nerve stimulation. [↑](#footnote-ref-4)
5. Other serious events are not system, device, lead or procedure related. They include events which required an intervention or hospitalization. [↑](#footnote-ref-5)
6. All procedure related non-serious events occurred up to 30 days after implant and include events without intervention: diaphragmatic nerve stimulation with device programming, lead dislodgment with device reprogramming, pocket hematoma, coronary sinus dissection, thrombosis, subclavian artery punction, pain, dyspnea, moderate pneumothorax, atrial fibrillation, elevated LV lead threshold, dizziness, elevated lead impedance, lead repositioning during implant procedure. [↑](#footnote-ref-6)
7. System and device related non-serious events include events with device reprogramming. [↑](#footnote-ref-7)
8. Lead related non-serious events include events without intervention: lead configuration reprogramming, diaphragmatic nerve stimulation with device programming, atrial flutter, external interferences, oversensing with device reprogramming, undersensing with device reprogramming, elevated lead impedance, elevated lead threshold with device reprogramming, loss of capture with device reprogramming, lead dysfunction [↑](#footnote-ref-8)
9. Other non-serious events are not system, device, lead or procedure related. They include events which required only medication change, no intervention and no hospitalization. [↑](#footnote-ref-9)
